# Deregulation of Factor H by Factor H-Related Protein 1 Depends on Sialylation of Host Surfaces

**DOI:** 10.3389/fimmu.2021.615748

**Published:** 2021-02-25

**Authors:** Arthur Dopler, Selina Stibitzky, Rachel Hevey, Marco Mannes, Mara Guariento, Britta Höchsmann, Hubert Schrezenmeier, Daniel Ricklin, Christoph Q. Schmidt

**Affiliations:** ^1^ Institute of Pharmacology of Natural Products and Clinical Pharmacology, Ulm University, Ulm, Germany; ^2^ Department of Pharmaceutical Sciences, University of Basel, Basel, Switzerland; ^3^ Institute of Transfusion Medicine, University of Ulm, Ulm, Germany; ^4^ Institute of Clinical Transfusion Medicine and Immunogenetics Ulm, German Red Cross Blood Transfusion Service and University Hospital of Ulm, Ulm, Germany

**Keywords:** complement system, factor H related protein 1, FHR-1, factor H (FH), FH deregulation, sialic acid

## Abstract

To discriminate between self and non-self surfaces and facilitate immune surveillance, the complement system relies on the interplay between surface-directed activators and regulators. The dimeric modulator FHR-1 is hypothesized to competitively remove the complement regulator FH from surfaces that strongly fix opsonic C3b molecules—a process known as “deregulation.” The C-terminal regions of FH and FHR-1 provide the basis of this competition. They contain binding sites for C3b and host surface markers and are identical except for two substitutions: S1191L and V1197A (i.e., FH “SV”; FHR-1 “LA”). Intriguingly, an FHR-1 variant featuring the “SV” combination of FH predisposes to atypical hemolytic uremic syndrome (aHUS). The functional impact of these mutations on complement (de)regulation, and their pathophysiological consequences, have largely remained elusive. We have addressed these questions using recombinantly expressed wildtype, mutated, and truncated versions of FHR-1 and FH. The “SV” to “LA” substitutions did not affect glycosaminoglycan recognition and had only a small effect on C3b binding. In contrast, the two amino acids substantially affected the binding of FH and FHR-1 to α2,3-linked sialic acids as host surfaces markers, with the S-to-L substitution causing an almost complete loss of recognition. Even with sialic acid-binding constructs, notable deregulation was only detected on host and not foreign cells. The aHUS-associated “SV” mutation converts FHR-1 into a sialic acid binder which, supported by its dimeric nature, enables excessive FH deregulation and, thus, complement activation on host surfaces. While we also observed inhibitory activities of FHR-1 on C3 and C5 convertases, the high concentrations required render the physiological impact uncertain. In conclusion, the SV-to-LA substitution in the C-terminal regions of FH and FHR-1 diminishes its sialic acid-binding ability and results in an FHR-1 molecule that only moderately deregulates FH. Such FH deregulation by FHR-1 only occurs on host/host-like surfaces that recruit FH. Conversion of FHR-1 into a sialic acid binder potentiates the deregulatory capacity of FHR-1 and thus explains the pathophysiology of the aHUS-associated FHR-1 “SV” variant.

## Introduction

The complement system is an essential component of innate immunity and consists of a cascade of soluble plasma proteins and several cellular receptors (1). Its activation leads to the opsonization of particles for clearance by phagocytosis or by cell lysis, and signaling through the release of anaphylatoxins. Central to the complement cascade is the formation of C3 convertases, large enzyme complexes that cleave complement component C3 into the anaphylatoxin C3a and the opsonin C3b, which can covalently bind to the activating surface. Surface-bound C3b can form a C3 proconvertase when it associates with factor B (FB), which is then cleaved by factor D (FD) to yield the functional bimolecular C3bBb complex, i.e., the C3 convertase of the alternative pathway (AP). The assembly of AP convertases, when left unchecked, creates an amplification loop, which is responsible for up to 80% of total complement activation (2). To avoid collateral damage when the complement cascade is triggered, host cells are equipped with preformed mediators of defense (3).

Among the most important regulators protecting host surfaces are the soluble regulators factor H (FH) and its splice variant factor H-like protein 1 (FHL-1) (4), which are composed of 20 and 7 complement control protein (CCP) domains, respectively (5–7). They contain identical N-terminal regulatory sites (CCP1-4), which bind and regulate complement convertases *via* decay accelerating (DAA) or cofactor activity (CA) (8). Moreover, they share a glycosaminoglycan (GAG) recognition site (CCP7) that can facilitate host surface binding (9, 10). However, the most critical site for self-recognition and self-protection is located on the two C-terminal domains of FH (CCP19-20), which are absent in FHL-1. While CCP7 and CCP20 bind GAGs albeit with slightly different selectivity (11), the C-terminal CCP20 is the only site in FH that specifically recognizes sialic acid moieties on host surfaces (12, 13).

Alongside FH and FHL-1, the FH family is also comprised of five FH-related proteins (FHR-1 to FHR-5). FHRs do not contain domains that are homologous to the convertase-regulating N-terminus of FH/FHL-1, yet show strong homology to FH’s recognition domains CCP7 and CCP18-20. FHR-5 is unique as the only FHR that contains domains with homology to FH CCPs 10-14 (14). Given the absence of convertase-binding domains, FHRs were initially considered to lack complement regulatory function (15). More recently, various specialized functions have been described in literature, including activities as decoy for FH-recruiting pathogens that recruit FH for complement evasion (reviewed in (16)), as negative regulators or, to the contrary, as enhancers of complement activation *via* “deregulation” (reviewed in (14)). Early studies investigating FHRs focused on the most abundant FHR, i.e., FHR-1, which was reported to circulate at approximately 40–100 µg/ml in human plasma (15, 17). A recent study utilizing a panel of tailor-made detection antibodies reported a lower FHR-1 plasma concentration of approximately 10–15 µg/ml (351 nM) (18). Two FHR-1 molecular weight variants exist, which both comprise five CCP domains but differ in their number of N-linked carbohydrates (one *versus* two sites, yielding a 37 or 42 kDa plasma protein, respectively) [reviewed in (19)]. Additionally, two genetic variants of FHR-1 exist: FHR-1*A and FHR-1*B (20). Whereas CCP3 of FHR-1*B is identical to CCP18 of FH, the CCP3 domain of the FHR-1*A variant differs in three amino acids. In both variants, CCP4-5 of FHR-1 is almost identical to FH CCP19-20 except for two amino acids which differ between CCP5 (L290, A296) and FH CCP20 (S1191, V1197).

It is generally accepted that FHR-1 forms homo- or heterodimers *via* its two N-terminal domains, which increases its avidity for C3b and C3d (21, 22). However, the functional role of FHR-1 and its structure-function relationship is still controversial. While some studies have found no complement inhibitory functions (15, 23), others have reported that FHR-1 inhibits C5 convertases (17, 24). Another set of studies demonstrated that FHR-1 competes with FH for binding to C3b and various cell surfaces, potentially displacing FH (termed “deregulation”). On both host (e.g., human endothelial cells) and foreign cells (guinea pig erythrocytes), FHR-1 mediated deregulation of FH in human serum was demonstrated (21, 25). The potential impact of FHR-1 on the survival of microbes is interesting but functional studies are scarce, especially those involving human serum with a full set of active complement components (16). Hence, given the discrepancy amongst literature reports, it cannot be generally stated whether FHR-1 favors deregulation of FH and on which cell surfaces this effect may be functionally relevant.

The inconclusiveness of the functional role of FHR-1 is contrasted by its proven association with different kidney and eye diseases [reviewed in (19)]. Recently, a mutation in the *CFHR1* gene was reported, which encodes an FHR-1 variant containing the L290S and A296V substitutions, thereby rendering CCP5 of FHR-1 identical to the host surface recognition domain CCP20 of FH (26). Individuals affected by this mutation presented with a severe form of atypical hemolytic uremic syndrome (aHUS). The disease mechanism was functionally explained by the mutant FHR-1 protein being a very strong competitor of FH for binding to cell surfaces. It was speculated that conversion of the FHR-1 C-terminus into the FH C-terminus transformed FHR-1 into a molecule that binds sialic acids and, therefore, competes more effectively with FH than wild-type FHR-1.

In order to prove this hypothesis and further elucidate structure-function relationships of FHR-1, we have expressed several mutant and truncated versions of FH and FHR-1. We have characterized these proteins in both binding assays and complement activation assays in serum utilizing different foreign and human cells. The results help to clarify under which conditions FHR-1 either activates or inhibits the complement cascade and sheds light on the underlying mechanisms.

## Materials and Methods

Some of the methods not listed here are described in **Supplementary Material**.

### Human Blood Components

Fresh red blood cells (RBCs) from paroxysmal nocturnal hemoglobinuria (PNH) patients under Eculizumab treatment or from healthy donors were used with approval from the Ethical Committee of Ulm University. Sera were collected in VACUETTE/S-Monovette serum collection tubes, aliquoted, frozen in liquid nitrogen and stored at −80˚C. Standardized normal human serum (NHS) and FI-depleted serum were obtained from CompTech.

### Proteins

Unless otherwise stated, complement proteins such as FH, C3, C3b, C5, FB, and FD were purchased from CompTech as plasma-purified proteins. Complement receptor 1 (CR1) CCP1-3, FHR-1, the complement receptor of the immunoglobulin family (CRIg), the mutant FHR-1(SV), the C-terminal FH fragments FH19-20 and FH18-20 and their mutated versions FH18-20(LV), FH18-20(SA), and FH18-20(LA) were recombinantly expressed in *Pichia pastoris* as previously described (27–29), with minor modifications. OmCI (Coversin) with a C-terminal his-tag was expressed in *Escherichia coli* and purified as described previously (30). All proteins were stored in PBS except for FHR-1 and FHR-1(SV), which were dissolved in glycine buffer (20 mM glycine, 150 mM NaCl, pH 10.5). FHR-1 and FHR-1(SV) were pre-diluted from stock solution into PBS to the concentration required for the specific assay; for both constructs, dimer concentrations are indicated. The quality of recombinantly expressed proteins was assessed by sodium dodecyl sulfate polyacrylamide gel electrophoresis (SDS-PAGE): 2 µg of each protein were loaded under both reducing and non-reducing conditions on a 4-12% Bis-Tris gradient gel (NuPAGE, Thermo Fisher Scientific), which was then stained with Coomassie Brilliant Blue R250 (Sigma-Aldrich). The protein ladder Page Ruler Plus prestained 10-250 kDa (Thermo Fisher Scientific) was loaded as reference.

### Heparin Affinity Chromatography

Heparin affinity for all produced proteins was tested on a HiTrap™ Heparin HP column (GE Healthcare). Samples containing 0.5 mg of protein were dissolved in 5 ml PBS (pH 7.4) to a final concentration of 0.1 mg/ml and loaded on the column equilibrated in PBS. Each protein was eluted by a gradient, over 10 column volumes, from PBS to PBS supplemented with 0.5 M NaCl.

### Endothelial Cell Opsonization Assay

Human microvascular endothelial cells (HMEC-1; ATCC) were cultured and the assay was performed as previously described (25) with increasing amounts (0.3-9.6 µM) of the different FH18-20 and FHR-1 analytes. Controls included 40% normal human serum (NHS) and 40% heat-inactivated serum (HIS), each diluted into PBS. Since FHR-1 and FHR-1(SV) were stored in 20 mM glycine buffer containing 150 mM NaCl (pH 10.5), a further control was prepared mixing NHS with the same concentration of glycine buffer present in the sample containing 9.6 µM of FHR-1(SV), to exclude any influence of this buffer on complement activation.

### Yeast Opsonization Assay

The yeast opsonization assay was performed as previously described (25), except that the final serum content was reduced from 40 to 5% and the final concentration of Mg-EGTA was 5 mM.

### Erythrocyte Binding Assay

Fresh human blood from healthy donors was collected in EDTA pre-dosed blood collection tubes. After centrifugation of a 200 µl cell suspension, the plasma was discarded and the cells were washed four times with 10 ml PBS and suspended to the initial volume. Next, 100 µl RBCs were mixed with 100 µl 20 mM Mg-EGTA, inhibitors (Eculizumab, OmCI, FH19-20 at 0.5, 0.5, and 10 µM final concentration, respectively), 160 µl factor I-depleted serum (40% final serum content, PBS for non-opsonized cells) and PBS to a final volume of 400 µl and incubated for 1 h at 37°C. The cells were washed once with 1 ml PBS after incubation and the opsonization process was repeated. After opsonization, cells were washed two times with 200 µl 20 µM FH19-20 to displace any remaining FH and three times with 1 ml PBS. Then, the cell suspension volume was adjusted to 200 µl, distributed to fresh tubes, and incubated with 50 U neuraminidase or PBS for 1 h at 37°C. Subsequently, a 10 µl aliquot of the untreated or desialylated cells was incubated with 50 µl of a 1:100 diluted FITC-labeled mouse anti-human C3 antibody (Cedarlane; clone: 7C12) for 30 min at room temperature and analyzed with flow cytometry after two wash steps with 1 ml PBS to control for proper C3b opsonization. If the measured C3b opsonization signal (median fluorescent intensity, MFI) on RBCs was not at least sixfold higher than non-opsonized cells, an additional opsonization process was applied. Having confirmed high opsonization levels, 10 µl cell suspensions were incubated with 10 µl of either analyte solution (0.75 µM final FH concentration with or without FHR-1 at 0.38, 0.75, or 7.5 µM) or PBS for 30 min at room temperature and then washed twice with 1 ml PBS. In order to detect FH, the cells were incubated with a mouse anti-human FH antibody (Quidel; clone: 131X). After a 10 min incubation the cells were washed twice with 1 ml PBS, incubated with an APC-labeled goat anti-mouse antibody (Jackson ImmunoResearch) for 10 min, and then again washed twice with 1 ml PBS. Finally, the cells were analyzed using a BD FACSVerse flow cytometer with MFIs calculated from the resulting histograms. The MFI values were normalized to the signal obtained from C3b opsonized cells treated with FH.

### PNH Erythrocyte Hemolysis Assay

The hemolysis assay on PNH erythrocytes was performed as previously described (25), with minor modifications. The final concentration of Mg-EGTA was 5 mM (instead of 6.25 mM). For experiments using desialylated erythrocytes, the cell suspension was treated with 50 U neuraminidase (New England Biolabs) instead of 36 U.

### Sheep Erythrocyte Hemolysis Assay

Sheep erythrocytes were supplied in Alsever’s solution (Fiebig-Nährstofftechnik) and washed with PBS. Then 10 µl of cell suspension (~ 1.3 x 10^9^ cells/ml) were mixed with 20 µl NHS, 10 µl of analyte solution (protein concentration from 0.3 to 9.6 µM), and 10 µl Mg-EGTA dissolved in PBS to prevent classical pathway activation (final serum and Mg-EGTA concentrations: 40% and 5 mM, respectively). The reactions were incubated for 30 min at 37°C and stopped by adding 150 µl ice-cold PBS supplemented with 5 mM EDTA. Afterward, the samples were spun down and the absorbance of the supernatants was measured at 405 nm.

Reaction controls included NHS, HIS, and the highest concentration of glycine buffer used in the samples. An additional control was prepared by mixing NHS and PBS supplemented with 5 mM EDTA. Hemolysis was calculated as the quotient of measured absorption of the sample and total lysis in water (100% reference).

### Rabbit Erythrocyte Hemolysis Assay

Rabbit erythrocytes were supplied in Alsever’s solution (Fiebig-Nährstofftechnik) and washed with PBS. A mixture of 10 µl of cell suspension (~ 1.3 x 10^9^ cells/ml), 10 µl of analyte solution (protein concentration from 0.6 to 9.6 µM), and 20 µl NHS diluted in PBS and supplemented with Mg-EGTA (final serum and Mg-EGTA concentrations: 15% and 5 mM, respectively) was incubated for 30 min at 37°C. The reaction was then stopped by adding 120 µl ice-cold PBS supplemented with 5 mM EDTA. Afterward the samples were spun down and the absorbance of the supernatants was measured at 405 nm.

The same reaction controls described for the sheep erythrocyte hemolysis assay were included.

Hemolysis was calculated as the quotient of measured absorption of the sample and total lysis in water (100% reference).

### Binding Affinity to C3b

The affinity of FHR-1, FHR-1(SV), FH18-20, FH18-20(LV), FH18-20(SA), and FH18-20(LA) to C3b was measured with surface plasmon resonance (SPR) spectroscopy according to previously described methods (31, 32) on a Reichert SPR7500DC SPR spectrometer (Reichert Technologies), setting the temperature to 25°C and the flow rate to 25 µl/min. Two different methods were used to immobilize C3b: biotin and amine coupling.

After incubation with 1mM DTT at room temperature and buffer exchange, C3b was biotinylated with EZ-Link Maleimide-PEG_2_-Biotin (Thermo Fisher Scientific), according to the manufacturer´s instructions. Approximately 1,500 response units (RUs) of biotinylated C3b were then immobilized onto a streptavidin chip (SAP; XanTec bioanalytics), which had been previously conditioned and washed according to the manufacturer’s recommendations.

For the amine coupling, a carboxymethyldextran hydrogel biosensor chip (CMD500M; XanTec bioanalytics) was conditioned and washed according to the manufacturer’s recommendations and then approximately 7,250 RUs of C3b were covalently attached to one of the flow cell surfaces.

Protein series (1 in 2 dilution) were prepared in running buffer (PBS containing 0.005% Tween20) in the following ranges: FH18-20 and mutants thereof were injected at 0.16–40 µM on the SAP chip and 0.63–40 µM on the CMD500M chip; FHR-1 and FHR-1(SV) were injected at 0.04–10 µM on both chips. After injection of analytes for 2.5 min, buffer was flowed over the chips and dissociation was observed for 5 min. For regeneration, 1 M NaCl was injected for 0.5 min. Only reference-subtracted sensorgrams are shown. Plots of response at steady state *versus* concentration were used for calculation of equilibrium dissociation constants *K*_D_ (TraceDrawer software, 1:1 steady-state affinity model).

### Binding Affinity to Sialic Acids

The binding of FHR-1, FHR-1(SV), FH18-20, FH18-20(LV), FH18-20(SA), FH18-20(LA), and FH19-20 to different sialic acid moieties was investigated by SPR. Approximately 150 RUs of two different biotinylated sialic acids, 3´-sialyl-*N*-acetyllactosaminide (α2,3-linked sialic acid; order number: OS31042) and 6´-sialyl-*N*-acetyllactosaminide (α2,6-linked sialic acid; OS31043) (Carbosynth), were coupled to a streptavidin chip (SAP; XanTec bioanalytics), previously conditioned and washed according to the manufacturer’s recommendations. For the binding experiments, a 1 in 2 dilution series of each analyte was flowed over the chip at the same concentrations as for the C3b binding assay on the SAP chip, FH19-20 was injected at 160–0.16 µM. Analytes were injected for 2.5 min and afterward dissociation was observed under buffer flow for 5 min. For regeneration, 1 M NaCl was injected for 0.5 min. All experiments were performed at 25°C and a flow rate of 25 µl/min.

### Inhibition of AP C3 Convertase Formation

The inhibition of AP C3 convertase formation was tested by SPR. Approximately 6,000 RUs of C3b were immobilized by standard amine coupling on a CMD500M chip (XanTec bioanalytics), which had been previously conditioned and washed according to the manufacturer’s recommendations. The AP C3 convertase signal alone was determined by injecting a mixture of FB (600 nM) and FD (100 nM) for 3 min and observing the natural C3 convertase decay for 5 min. In each experiment either the analyte alone (FH18-20, FH18-20(LA), FHR-1, or FHR-1(SV), all at 9.6 µM) or a mixture of the analyte (9.6 µM), FB (600 nM), and FD (100 nM) was injected for 3 min and the natural C3 convertase decay was observed for 5 min. After every injection, 1 µM recombinant CR1 CCP1-3 was flowed over the chip for 0.5 min to remove the remaining convertases by accelerating the decay. For regeneration, 1 M NaCl was injected for 0.5 min. All experiments were performed at 25°C in PBS containing 0.005% Tween20 and 1 mM MgCl_2_ at a flow rate of 25 µl/min. Finally, the TraceDrawer software was used to subtract the C3b binding signal of the analyte alone from the signal obtained for the analyte/convertase mixture.

### Inhibition of C3 Binding to C3b

SPR was used to evaluate if FH18-20, FH18-20(LA), FHR-1, or FHR-1(SV) inhibit C3 binding to C3b. Approximately 2,300 RUs of biotinylated C3b (prepared as previously described) were immobilized on a streptavidin chip (SAP; XanTec bioanalytics), previously conditioned and washed according to the manufacturer’s recommendations. First, C3 binding to C3b was measured by injecting 1 µM C3 for 2 min and dissociation was observed for 5 min. Then, the binding signal to C3b was determined for each construct by injecting each protein at a concentration 10-fold higher than its respective measured K_D_ [100 µM FH18-20, 60 µM FH18-20(LA), 6 µM FHR-1, and 15 µM FHR-1(SV)] for 2 min, followed by 5 min dissociation monitoring. Finally, C3 (1 µM) and each construct (at a concentration 10-fold higher than the K_D_) were co-injected for 2 min and dissociation was observed for 5 min. For regeneration, 1 M NaCl and buffer were flowed for 0.5 and 2 min, respectively, after each protein injection. All experiments were performed in duplicate, at 25°C in PBS containing 0.005% Tween20 and at a flow rate of 25 µl/min.

### Inhibition of C5 Binding to C3b

Inhibition of C5 binding to C3b was performed by SPR in a similar manner as described for the inhibition of C3 binding to C3b, with only a few changes. For this experiment, 1,500 RUs of biotinylated C3b were immobilized on the streptavidin chip and a concentration of 0.15 µM C5 was used instead of C3. Additionally, the single mutants of FH18-20 were also tested using concentrations 10-fold higher than their K_D_ values [100 µM FH18-20(SA) and 60 µM FH18-20(LV)]. As a control, the same measurements were performed with 100 µM FH19-20.

### Saturation Transfer Difference Nuclear Magnetic Resonance Spectroscopy

NMR spectra were recorded at 283 K on a Bruker Avance III 500 MHz NMR spectrometer equipped with a TXI room temperature probe head. Spectra were acquired and processed with Topspin 3.5 software (Bruker). Samples were prepared in 3 mm tubes and contained 5 mM glycan (3’-sialyllactose or 6’-sialyllactose referred to as α2,3-linked or α2,6-linked sialic acids, respectively; Carbosynth) and 50 µM recombinant FH protein in deuterated buffer. Glycans were added from a 40 mM stock solution in D_2_O. Protein samples were prepared by buffer exchange into deuterated NMR buffer (20 mM potassium phosphate, pH 7.4, 150 mM NaCl in D_2_O) using 3 kDa MWCO regenerated cellulose centrifugal filters (Amicon Ultra 0.5 ml, Merck Millipore). STD NMR spectra were collected using a train of Gaussian-shaped pulses with a total saturation time of 3 s, off- and on-resonance irradiation frequencies of −30 ppm and 7.3 ppm, respectively, and a total relaxation delay of 4 s. A 30 ms continuous-wave spin-lock pulse was used for the suppression of residual protein signals, and the water signal was suppressed using a 2.4 ms 180° shaped pulse (concomitantly reducing the Gal and Glc anomeric proton resonances). 4k scans were collected for each experiment, and spectra were referenced to the NHAc methyl signal at 298 K as a standard (3’SL: 2.00 ppm; 6’SL: 2.02 ppm). Absolute STD values were determined and are reported as a % of the off-resonance signal intensity (specific resonances used have been indicated). Samples containing glycan but lacking protein were used to collect 1D reference spectra and perform control experiments to confirm that no direct irradiation of ligand occurs under the conditions used for protein-containing STD experiments. Glycan structures were solved in D_2_O and assignments performed using standard 2D experiments (COSY, HSQC) with spectra referenced to a CH_3_OH internal standard (δH: 3.34 ppm, δC: 49.50 ppm).

### Intrinsic Tryptophan Fluorescence Assay

Intrinsic tryptophan fluorescence was measured using a nano-differential scanning fluorimetry instrument (Prometheus NT.48, NanoTemper) by exciting samples at 285 nm and measuring fluorescence emission at 330 and 350 nm. Measurements were taken at 293 and 310 K, using 10 µl sample volume per measurement in standard grade capillaries. Each concentration was measured in triplicate, and the full experiment run in duplicate to ensure reproducibility. Each sample contained 25 µM FH18-20 with the respective glycan concentration (9.8 µM–40 mM of 3’-sialyllactose/6’-sialyllactose; Carbosynth) in 50 mM HEPES, pH 7.4, 150 mM NaCl, 0.005% Tween 20, and 3 mM EDTA.

## Results

### FH Mutations S1191L and V1197A Impair Neither C3b Nor Heparin Binding

**Figure 1:**
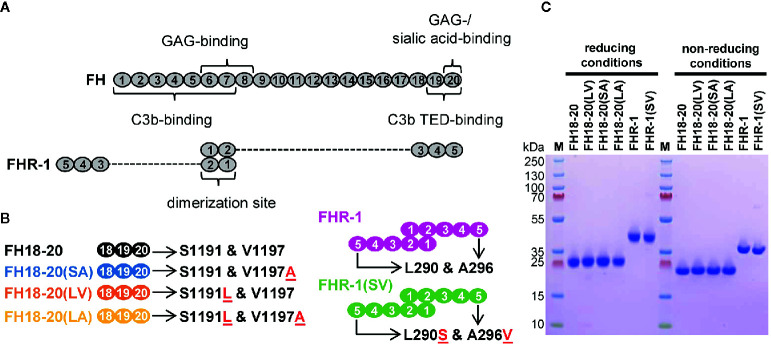
Schematic structure and sodium dodecyl sulfate polyacrylamide gel electrophoresis (SDS-PAGE) analysis of the recombinantly expressed FH18-20 and FHR-1 variants used in this study. **(A)** Schematic structure of FH and FHR-1. Every oval represents a CCP domain. The first two CCP domains of FHR-1 (dimerization site) share 42 and 34% identity with the corresponding CCPs in FH (CCPs 6 and 7). FHR-1 domains 3, 4, and 5 share 100, 100, and 98% identity with CCPs 18, 19, and 20 in FH. Previously reported binding sites are shown. **(B)** Schematic representation of the proteins used in this study. Mutations are indicated by the red underlined one letter code amino acid found in the mutant. The name of each construct, except for the wild type species, reports in brackets the amino acids corresponding to positions 1,191 and 1,197 (for FH) or 290 and 296 (for FHR-1). For clarity, the color used to depict each construct is maintained throughout the text and figures. **(C)** SDS-PAGE analysis of recombinantly expressed FH18-20 and FHR-1 variants. 2 μg of each protein were loaded under both reducing and non-reducing conditions on a 4-12% Bis-Tris gradient gel, then stained with Coomassie Brilliant Blue R250.

To gain insights into the function of wildtype FHR-1 and the aHUS-associated FHR-1 variant (L290S and A296V), we expressed FHR-1*B (wild-type and mutated sequences) in addition to constructs comprising only the last three CCP domains of FHR-1*B and FH (**Figure 1**). To study the functional impact of each aHUS-linked mutation individually, single amino acid mutant constructs were also prepared. All constructs (schematically represented in **Figures 1A, B**) were recombinantly expressed in the yeast *P. pastoris* and prepared to a high level of purity (**Figure 1C**).

**Figure 2:**
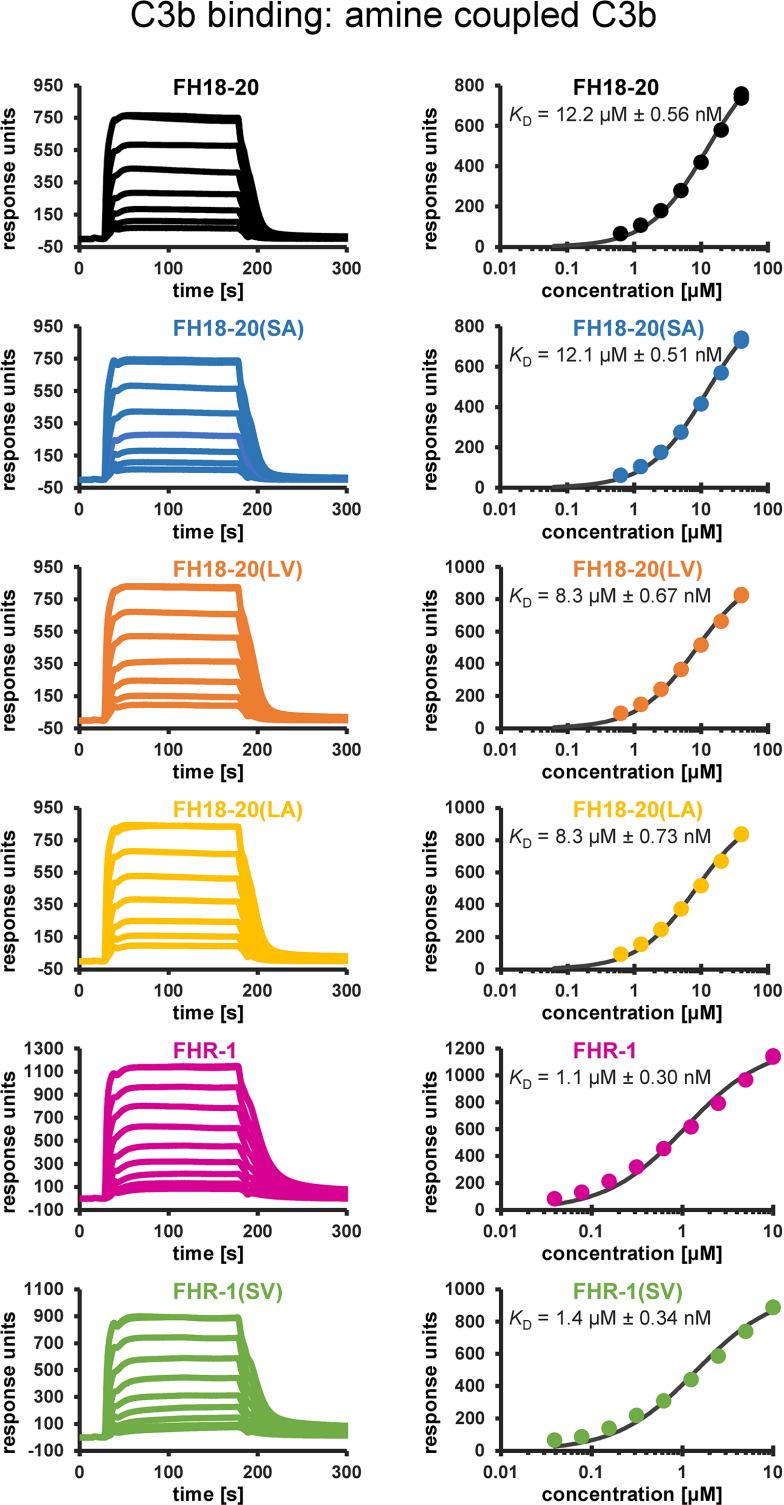
Binding affinity to amine-coupled C3b assessed by SPR. SPR sensorgrams (not normalized to m.w.) of C3b binding and corresponding response–concentration plots with 1:1 steady-state affinity fits are shown. Recombinant FH18-20 and FHR-1 constructs were flowed over a carboxymethyldextran (CMD) biosensor chip with 7,250 RUs of amine-coupled C3b (left panels). The corresponding concentration–response plots with the extracted K_D_ values are shown in the right panels. Reference-subtracted sensorgrams are shown.

For all constructs, their affinity for C3b was determined by SPR using two different methods of C3b immobilization on the sensor chip surface (biotin- and amine-coupling). As previously reported, the affinity of FH18-20 for C3b was in the range of 9.9–12.2 µM. Consistent with the results published for corresponding mutations in the truncated FH fragment FH19-20 in Morgan et al. (28), the S1191L mutation in FH18-20(LV) slightly enhanced the affinity of FH18-20 for C3b (5.6–8.3 µM). In contrast, the V1197A mutation in FH18-20(SA) did not have a notable impact on *K*_D_ when compared to the wildtype construct (10.0-12.1 µM). The affinity of the double mutant FH18-20(LA) for C3b was in the range of 5.8–8.3 µM and therefore similar to that of the single mutant FH18-20(LV). The *K*_D_ of FHR-1 for C3b was in the range of 0.6–1.1 µM, while its double mutant FHR-1(SV) showed a slightly decreased affinity (1.4–1.5 µM) (**Figure 2**). Overall, the analytes exhibited similar relative affinities for C3b when they were randomly oriented (amine-coupling) *versus* being more physiologically oriented *via* the thioester bond (biotin-coupling) (**Supplementary Figure 1**).

**Figure 3:**
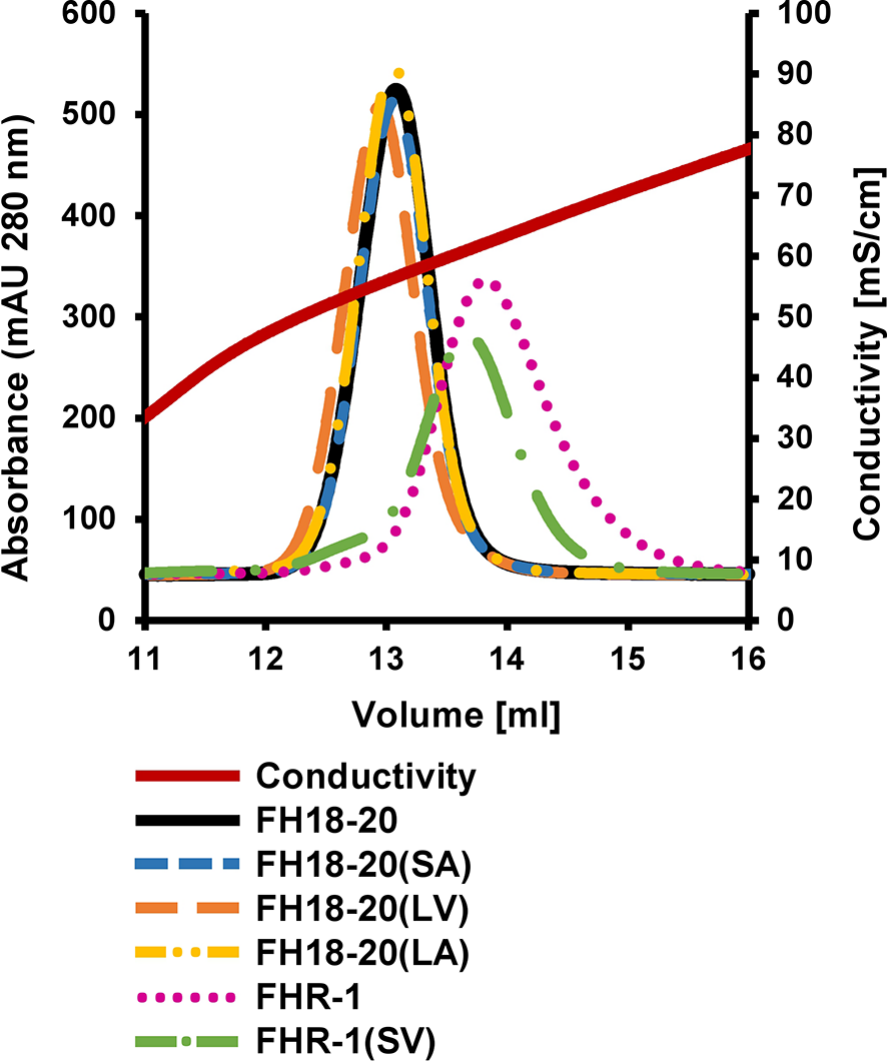
Heparin affinity chromatography. 0.5 mg of each protein were loaded onto a HiTrap heparin HP column after being diluted in PBS. Proteins were eluted with a linear salt gradient (0–0.5M NaCl) over 10 CV.

To evaluate if either of the mutations (S1191L or V1197A), which are also located in an FH GAG-binding site (*i.e.*, CCP20), affect the binding of FH18-20 for a model GAG, we performed affinity chromatography on a heparin column for all FH18-20 and FHR-1 constructs (**Figure 3**). All monomeric FH18-20 constructs [FH18-20, FH18-20(LV), FH18-20(SA) and FH18-20(LA)] eluted from the affinity resin at a similar conductivity (55.4–56.6 mS/cm, corresponding to 13.0–13.1 ml elution volume). The dimeric proteins FHR-1 and FHR-1(SV) both eluted later (61.0–62.2 mS/cm, corresponding to ∼ 13.7–13.8 ml elution volume), thus displaying a stronger binding for heparin. This is not unexpected due to the presence of two GAG-binding sites in the dimers. In summary, the presented results show that binding to neither C3b nor the model GAG heparin was substantially altered by FH mutations S1191L and V1197A.

### FH Mutation S1191L Abrogates Sialic Acid Binding

**Figure 4:**
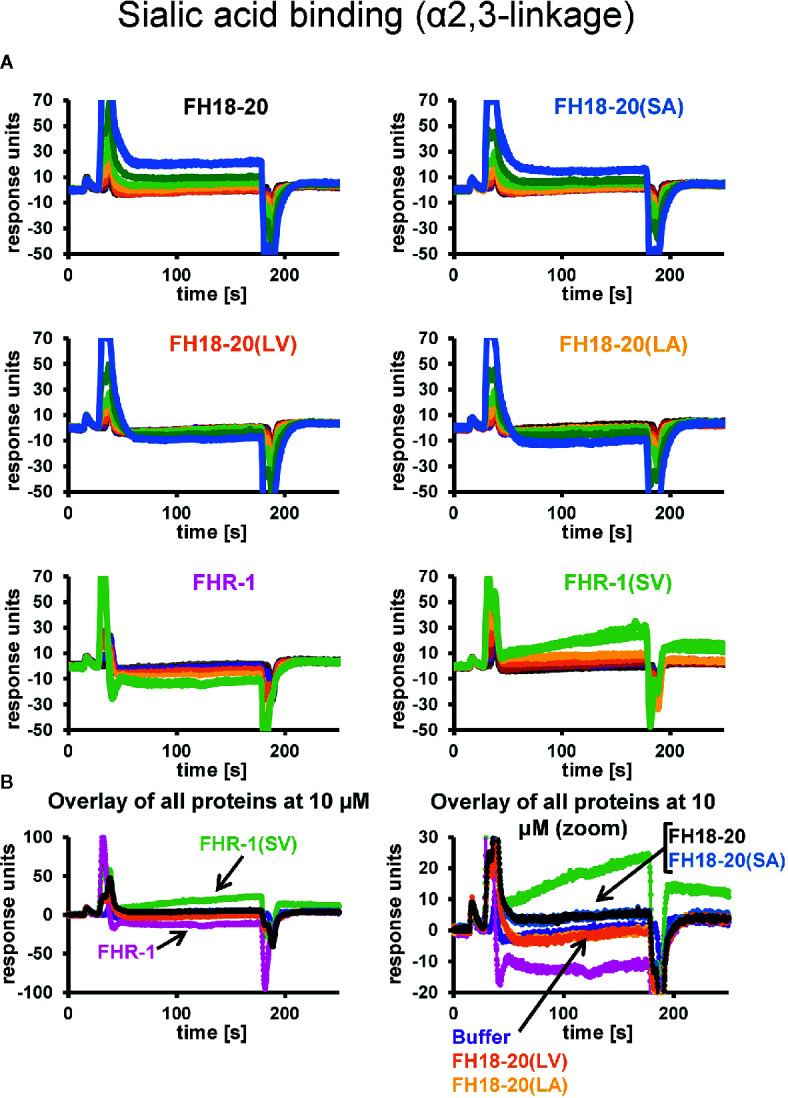
α2,3-Linked sialic acid affinity measurements by SPR. The affinities of FH18-20, FHR-1, and their mutant versions for α2,3-linked sialic acid were measured by SPR. All proteins were flowed over a streptavidin sensor chip where approximately 150 RUs of biotin-coupled α2,3-linked sialic acid had been immobilized. **(A)** 1:1 dilution series of each construct were prepared at concentrations ranging from 0.16 to 40 µM for FH18-20 and its mutants, and 0.04–10 µM for FHR-1 and FHR-1(SV). The corresponding binding curves are shown in blue (40 µM), dark green (20 µM), light green (10 µM), yellow (5 µM), orange (2.5 µM), red (1.25 µM), purple (0.63 µM), dark brown (0.31 µM), light brown (0.16 µM), gray (0.08 µM), and black (0.04 µM). **(B)** Overlay of binding responses to α2,3-linked sialic acid of all analytes at 10 µM.

**Figure 5:**
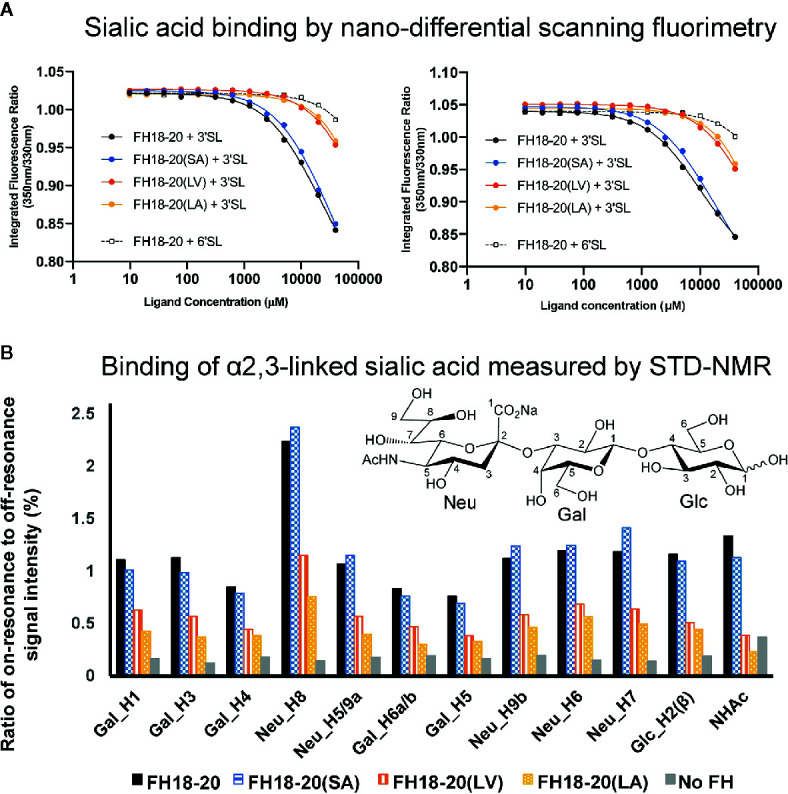
Assessment of the interaction between monomeric FH18-20 constructs and sialyllactose in solution. **(A)** Nano-differential scanning fluorimetry (nano-DSF) measurements. Determination of the intrinsic tryptophan fluorescence of wildtype, single, and double mutants of FH18-20 in the presence of either α2,3-linked sialic acid (3’SL) or α2,6-linked sialic acid (6’SL) was performed at 20°C (left panel) and 37°C (right panel). The different FH18-20 constructs (25 µM) were incubated with increasing amounts of sialylated ligand (9.8 µM–40 mM), and then excited at 285 nm and the fluorescence emission recorded at 330 and 350 nm. Concentrations were measured in triplicate and the full experiment run in duplicate to ensure reproducibility. **(B)** α2,3-Linked sialic acid affinity measurements by STD-NMR. α2,3-Linked sialic acid binding of FH18-20 and its mutational variants was determined by STD-NMR. The histograms report the ratio of on-resonance to off-resonance signal intensities (%) measured upon binding of FH18-20 constructs to different hydrogen atoms (H) of α2,3-linked sialyllactose. The order of signals along the x-axis reflects their relative chemical shifts in the NMR spectra. A chemical structure for α2,3-linked sialyllactose is provided for reference.

The prediction that a leucine at position 1,191 in FH would impair sialic acid binding by causing a steric clash with the ligand was previously formulated in a report describing the structure of the FH CCP19-20 in complex with a sialylated trisaccharide and the thioester-containing domain (TED) of C3b (12). To verify this hypothesis, we tested the binding of all FH18-20 and FHR-1 constructs to immobilized α2,3-linked sialic acid and α2,6-linked sialic acid by SPR. By flowing different concentrations of protein over the sensor chip, we could observe a similarly weak binding of FH18-20 and FH18-20(SA) to α2,3-linked sialic acid, while binding of FHR-1, FH18-20(LV), and FH18-20(LA) (all containing a leucine in position 290 or 1,191, for FHR-1 or FH18-20 constructs, respectively) was not detected (**Figure 4A**). The dimeric construct FHR-1(SV), containing two potential binding sites for sialic acids and thus enhancing avidity for the immobilized ligand surface, showed the strongest interaction for α2,3-linked sialic acid with a slow on- and off-rate (see **Figure 4B**). As expected, none of the tested constructs bound α2,6-linked sialic acid when injected at 10 µM concentration (**Supplementary Figure 2**). Probing a concentration series of FH18-20 and FH19-20 revealed that the affinity of CCP20 for immobilized α2,3-linked sialic acid is higher than 100 µM (**Supplementary Figure 2**); of note, the affinity values provided for these interactions are considered rough estimates as saturation was not reached, yet they indicate a low affinity in the high µM or low mM range. To corroborate and validate the SPR-derived results, we employed nanoDSF as a fluid phase method to assess the binding of FH constructs to α2,3 and α2,6-linked sialic acid (**Figure 5A**). Analogous to SPR, notably stronger binding to α2,3-linked sialic acid was observed for wildtype FH18-20 and the SA mutant as compared to the LV and LA mutants, thereby supporting that the S1191L mutation impacts sialic acid binding. As expected, monovalent interactions in solution were generally weaker when compared to the surface-based SPR method, with estimated affinities in the millimolar range. Finally, the binding of wildtype FH18-20 and its three mutant versions to sialic acids was also probed by saturation transfer difference nuclear magnetic resonance (STD NMR) spectroscopy (12). In agreement with SPR data, FH18-20 and the single mutant FH18-20(SA) bound α2,3-linked sialic acid with comparable strength (**Figure 5B**), and negligible binding to α2,6-linked sialic acid was observed for all proteins (**Supplementary Figure 3**). In contrast, constructs carrying the S1191L mutation, *i.e.*, the single and double mutants FH18-20(LV) and FH18-20(LA), showed strongly reduced binding to α2,3-linked sialic acid (**Figure 5B**).

Therefore, we experimentally confirmed the hypothesis that a serine-to-leucine substitution at position 1,191 in FH disrupts sialic acid binding, as predicted in Blaum et al. (12).

### Sialic Acid-Binding Constructs Compete with Serum FH on Self and Self-Like Surfaces

Next we investigated how the differences in sialic acid binding affect FH deregulation on self (human endothelial cells and RBCs) and self-like surfaces (sheep and guinea pig RBCs), which contain a layer of sialic acid that has been shown to be important for binding human FH (33–36).

**Figure 6:**
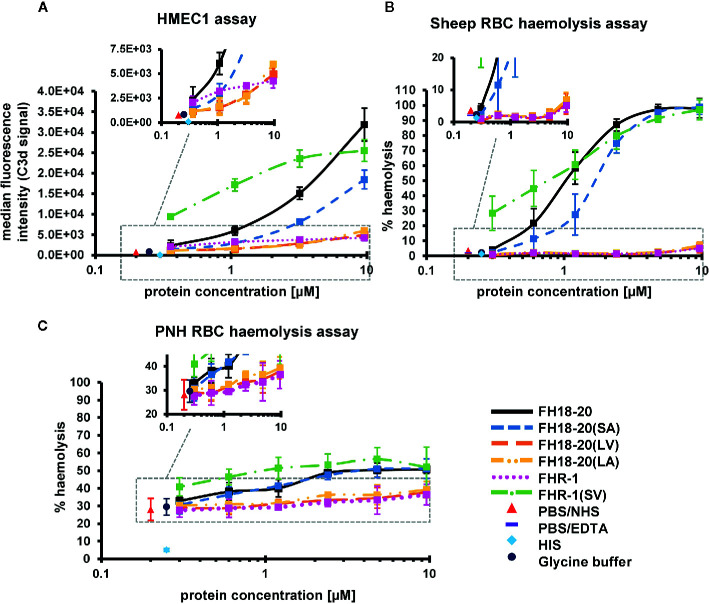
Functional cell assays on self and self-like surfaces. **(A)** Adherent human microvascular endothelial cells (HMEC-1) opsonization assay. HMEC-1 were incubated with normal human serum (NHS) in the presence of increasing amounts (0.36–9.6 µM) of the different FH18-20 and FHR-1 analytes. The cells were labeled with anti-C3d antibody and an APC fluorescence marker to measure levels of C3-opsonization. The mean of the MFI of three independent assays (shown in **Supplementary Figure 4**), each conducted in duplicate, is plotted *versus* the protein concentration in µM with standard deviation (SD). As controls, NHS and heat inactivated serum (HIS) were used, each mixed with PBS. As a further control NHS was mixed with the highest concentration of glycine buffer used in the sample containing 9.6 µM of FHR-1(SV). MDFIs were calculated with the software tool FlowJo (version 7.6.5). **(B)** Sheep erythrocytes hemolysis assay. Sheep erythrocytes were incubated with NHS in the presence of increasing amounts (0.3–9.6 µM) of the different FH18-20 and FHR-1 analytes. Lysis and subsequent release of hemoglobin was determined by UV absorbance at 405 nm. All values were normalized to lysis in water (100% lysis mark). The mean percentage of hemolysis of three independent assays, each conducted in duplicate, is shown with SD and plotted against the protein concentration in µM. Controls were included as described for the HMEC-1 opsonization assay. A fourth control was added, which consisted of NHS mixed with PBS containing 5 mM EDTA. **(C)** PNH erythrocytes hemolysis assay. PNH erythrocytes were incubated with NHS in presence of increasing amounts (0.3–9.6 µM) of the different FH18-20 and FHR-1 analytes. Lysis was determined as described for the sheep erythrocytes hemolysis assay. The mean percentage of hemolysis of three independent assays, each conducted in duplicate, is shown with SD and plotted against the protein concentration in µM. The same controls as in the HMEC-1 opsonization assay were included.

We first evaluated if our recombinant constructs were able to compete with serum FH on human microvascular endothelial (HMEC-1) cells, thus reducing physiological protection from AP-mediated attack. We found that all constructs carrying the leucine in position 290/1,191 [*i.e.*, FHR-1, FH18-20(LV) and FH18-20(LA)] competed very mildly with serum FH and that this effect was more visible at higher protein concentrations. Conversely, FH18-20, FH18-20(SA), and FHR-1(SV) competed more efficiently with serum FH, although the single mutant FH18-20(SA) was about half as active as the wild type FH18-20 (**Figure 6A**, primary data in **Supplementary Figure 4**).

**Figure 7:**
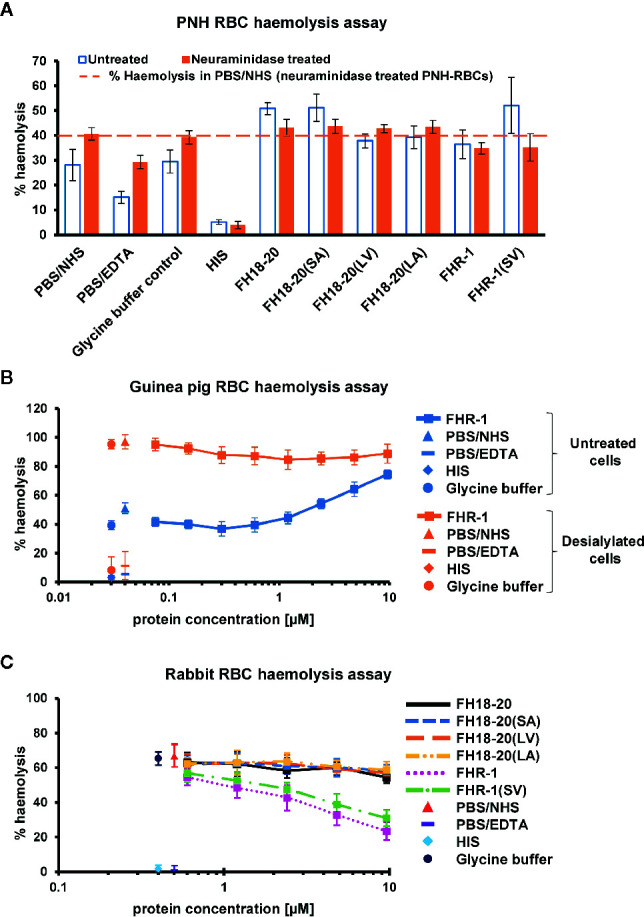
Functional cell assays on foreign and foreign-like surfaces. **(A)** Hemolysis assay of untreated and neuraminidase-treated PNH erythrocytes. Desialylated PNH erythrocytes were treated with neuraminidase before exposing them to serum. PNH erythrocytes were incubated with NHS in presence of 9.6 µM of the different FH18-20 and FHR-1 analytes. Lysis and subsequent release of hemoglobin was determined by UV absorbance at 405 nm. All values were normalized to lysis in water (100% lysis mark). The mean percentage of hemolysis of three independent assays, each conducted in duplicate, is shown (with SD) for untreated (white with blue border) and neuraminidase-treated (orange) PNH erythrocytes. As controls, NHS and HIS were used, each mixed with PBS. As a further control NHS was mixed with the highest concentration of glycine buffer used in the sample containing 9.6 µM of FHR-1(SV). A fourth control was added, which consisted of NHS mixed with PBS containing 5 mM EDTA. The dashed line indicates the hemolysis level in PBS/NHS for neuraminidase-treated PNH erythrocytes as reference. **(B)** Hemolysis assay of untreated and neuraminidase-treated guinea pig erythrocytes. Same assay as in **(A)**, but only a concentration series (9.6–0.1 µM) of FHR-1 as analyte. **(C)** Rabbit erythrocytes hemolysis assay. Rabbit erythrocytes were incubated with NHS in the presence of increasing amounts (0.3–9.6 µM) of the different FH18-20 and FHR-1 analytes. Lysis was determined as described for the neuraminidase-treated PNH erythrocytes hemolysis assay. The mean percentage of hemolysis of three independent assays, each conducted in duplicate, is shown with SD and plotted against the protein concentration in µM. The same controls were used as described for the neuraminidase-treated PNH erythrocytes hemolysis assay.

Similar results were obtained when hemolysis assays were performed on sheep RBCs, guinea pig RBCs or on RBCs from patients suffering from paroxysmal nocturnal hemoglobinuria (PNH). In all three cases, the stronger sialic acid binders (FH18-20, FH18-20(SA) and FHR-1(SV)) could compete better with serum FH, with FHR-1(SV) causing the highest level of hemolysis on PNH RBCs. The non-sialic acid-binding constructs instead showed much lower competition with serum FH on PNH and sheep RBCs but relatively strong competition for FHR-1 on guinea pig erythrocytes (**Figures 6B, C** and **7A, B** untreated cells and **Supplementary Figure 5**).

In conclusion, these data show that, although FHR-1 and the other non-sialic acid-binding constructs carrying a leucine in position 290/1,191 [FH18-20(LV) and FH18-20(LA)] were able to compete with serum FH on both self and self-like surfaces when applied at high concentration, the sialic acid-binding constructs were by far the most efficient deregulators.

### Dimeric Constructs Protect Foreign and Foreign-Like Surfaces at High Concentrations

To assess if deregulation occurs on different types of surfaces, we extended the analysis to foreign (rabbit RBCs and yeast cells) and foreign-like cells (desialylated PNH and guinea pig RBCs).

By removing terminal sialic acid residues, neuraminidase turns self and self-like surfaces such as PNH or guinea pig RBCs into foreign-like surfaces. A hemolysis assay performed on neuraminidase-treated PNH RBCs revealed that all monomeric constructs showed a very similar level of hemolysis as the PBS and glycine buffer controls, independent of their ability to bind sialic acid, thus excluding the possibility that any deregulation was occurring. Surprisingly, the dimeric constructs FHR-1 and FHR-1(SV), when given at very high concentration, were able to partially protect these cells from lysis (**Figure 7A**). In agreement with this observation, high concentrations of FHR-1 on neuraminidase-treated guinea pig RBCs also showed a slight decrease in hemolysis (**Figure 7B**).

**Figure 8:**
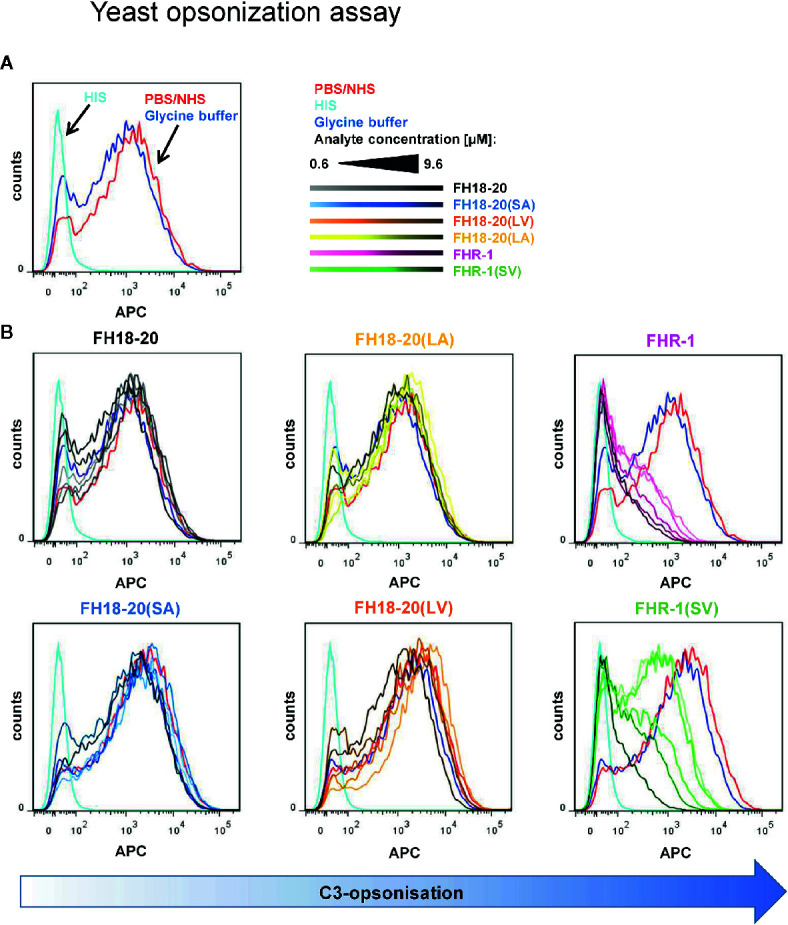
Yeast opsonization assay. Flow cytometric analysis of *Pichia pastoris* KM71H yeast cells after exposure to serum mixed with increasing amounts (0.6–9.6 µM) of the different FH18-20 and FHR-1 analytes. Cells were labeled with anti-C3d antibody and a secondary detection molecule with APC fluorescence marker to measure levels of C3-opsonization. As controls, NHS (red) and HIS (cyan), each mixed with PBS as analyte, and NHS mixed with the highest concentration of glycine buffer used in the sample containing 9.6 µM of FHR-1(SV) were used. **(A)** The controls without protein analytes are shown. **(B)** The histograms of each analyte series are shown. In each series the increasing concentration of the analyte is indicated by a corresponding color gradient. One representative of two independent experiments is shown (the second experiment is reported in the **Supplementary Figure 6**). FlowJo (version 7.6.5) was used as evaluation tool.

Moreover, no deregulation was observed on rabbit RBCs for any FH construct or the FHR-1 and FHR-1(SV) dimers. However, when the dimeric constructs were given at very high concentrations (> 1 µM), rabbit RBCs were protected from lysis (**Figure 7C**). Very similar results were obtained in a yeast opsonization assay, where all monomeric constructs led to comparable levels of C3 deposition, but the dimeric constructs substantially inhibited opsonization with C3b at high concentrations (**Figure 8** and **Supplementary Figure 6**).

We therefore conclude that deregulation was completely absent on the tested foreign and foreign-like surfaces, but also that dimeric FHR-1 constructs, at concentrations exceeding reported plasma levels, showed protection of non-sialylated cell surfaces, independent of their ability to bind sialic acid.

### Dimeric Constructs Interfere With Formation of the AP C3 Convertase When C3b Is Densely Deposited

In view of this unexpected finding, we investigated the mechanisms responsible for the protection of foreign and foreign-like surfaces observed for the dimeric constructs. Whereas it has previously been reported that FHR-1 can inhibit C5 convertases (17), the observed inhibition of C3b deposition on yeast cells in our study indicated that FHR-1 and FHR-1(SV) may already interfere with formation of the AP C3 convertase, thereby resulting in reduced opsonization.

**Figure 9:**
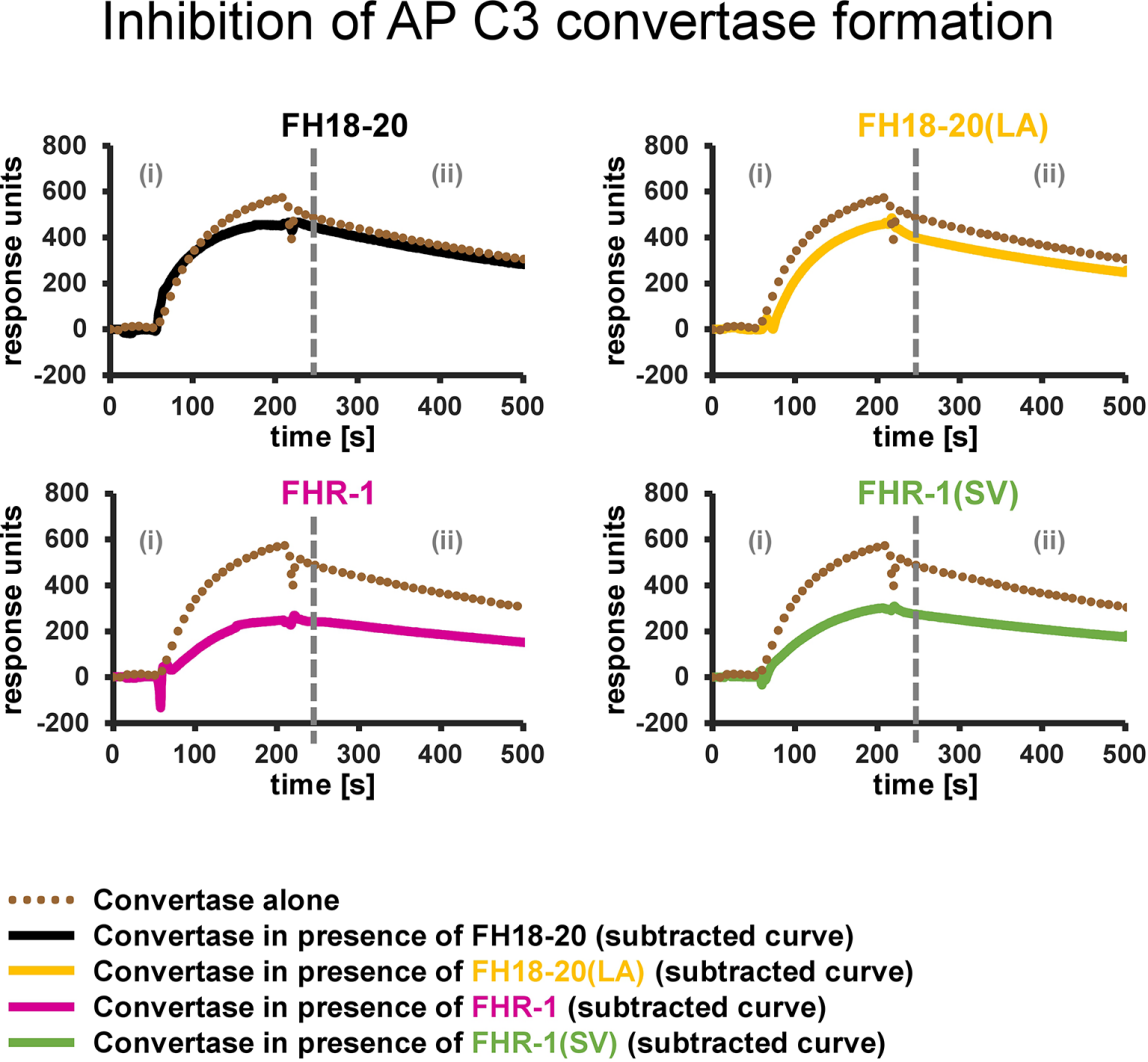
Inhibition of AP C3 convertase formation. Effect of FH18-20, FHR-1 and their double mutants on AP C3 convertase formation. In each panel two different sensorgrams are shown: the signal of the AP C3 convertase alone, obtained by injection of FB (600 nM) and FD (100 nM) over a CMD500M sensor chip with 6,000 RUs of amine-coupled C3b immobilized, is shown as a dotted brown line. The curves obtained subtracting the signal of the analyte alone (injected at a concentration 10 times higher than the measured K_D_ value) from that of the analyte/convertase mix (subtracted curves) are shown in solid black, yellow, pink, and green for FH18-20, FH18-20(LA), FHR-1, or FHR-1(SV), respectively. All shown sensorgrams are reference-subtracted. All sensorgrams, including the subtracted curves and their overlay, are reported in the **Supplementary Figure 7**.

To test this hypothesis, we used SPR to compare the formation of the AP C3 convertase on a sensor chip with densely deposited C3b, in presence and absence of FH/FHR-1 constructs (**Figure 9** and **Supplementary Figure 7**). Injection of a mixture of FB and FD on C3b resulted in the expected AP C3 convertase signal with slow association and dissociation phases. The monomeric proteins, FH18-20 and the double mutant FH18-20(LA), did not have a notable impact on convertase formation signals after the SPR responses were corrected for C3b-regulator binding. Conversely, when either of the FHR-1 or FHR-1(SV) dimers were co-injected at high concentration with FB and FD, the corrected curves were ~50% lower than those of the convertase alone. Therefore, a decrease in the formation of the AP C3 convertase could be detected.

In summary, the presented results indicate that FHR-1, independent of the aHUS-related mutation, reduces C3b opsonization of foreign and foreign-like cells by decreasing the formation of the AP C3 convertase on these surfaces.

### Dimeric Constructs Do Not Interfere with Binding of C3 to Densely Deposited C3b

In another experiment, we tested whether the dimers also had an obstructing effect on C3 binding to C3b. This would stop existing convertases from cleaving additional C3 into C3b, thus preventing further C3b opsonization.

This evaluation was performed by SPR on a streptavidin chip, where biotinylated C3b had been densely immobilized in a physiological orientation. By flowing a mixture of C3 and each of our constructs at a very high concentration over the C3b surface, we could establish that the resulting SPR responses corresponded to the sum of the signals by C3 alone and each individual construct alone (**Supplementary Figure 8**). We could therefore conclude that no inhibition of C3 binding to C3b took place neither in presence of the FH18-20 constructs nor the FHR-1 variants.

### Dimeric Constructs Inhibit Binding of C5 to Very Densely Deposited C3b

Several studies have shown that C5 binding to C3b is a prerequisite for C5 activation (37–41). Therefore, another possible explanation for the lower level of hemolysis observed on foreign and foreign-like surfaces in the presence of the dimeric constructs could be that these proteins are able to reduce C5 binding to C3b, thereby impairing C5b production and, thus, hindering the activation of the complement terminal pathway.

To verify this hypothesis, we tested the binding of C5 to immobilized C3b by SPR, in the presence and absence of our constructs, similar to that described for the C3 experiment in the previous paragraph.

No inhibition of C5 binding to C3b was observed when the monomers [FH19-20, FH18-20, FH18-20(LV), FH18-20(SA) and FH18-20(LA)] were co-injected with C5. However, the FHR-1 and FHR-1(SV) dimers did both cause inhibition of C5 binding to C3b, as can be inferred from the fact that the binding signals of the constructs alone almost overlap with those of the analyte/C5 mixtures (**Supplementary Figure 9**).

In conclusion, we present data showing that FHR-1 and its variant FHR-1(SV) equally inhibit C5 binding to C3b at very high concentrations, thus suggesting a further surface protection mechanism, whereby activation of the complement terminal pathway, and therefore cell lysis, may be reduced at non-physiological high concentrations of FHR-1.

### Competition of FHR-1 With FH for C3b Binding

**Figure 10:**
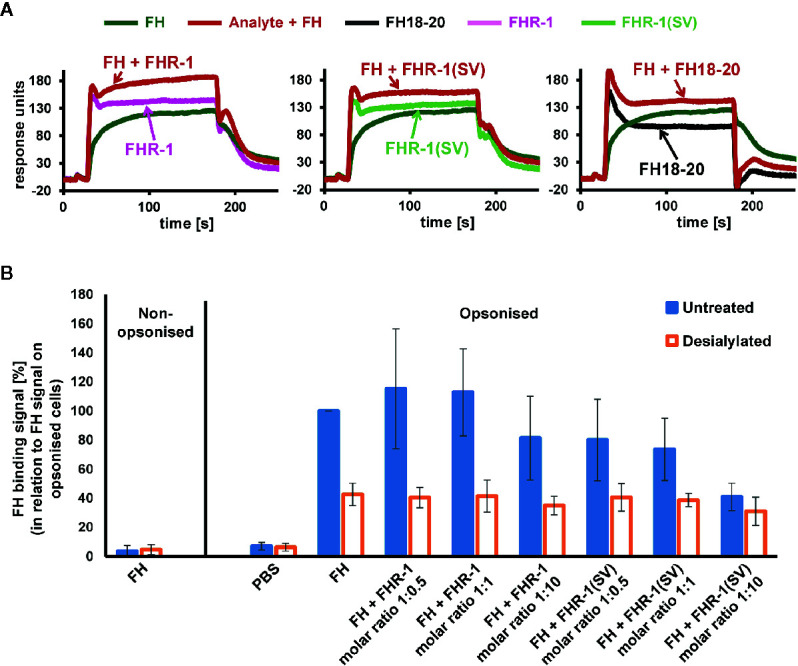
Competition of FH and FHR-1. **(A)** Assessment of competition between FH and FHR-1 on an SPR chip that was immobilized with approximately 430 RU of biotinylated C3b on a streptavidin sensor chip. Either FHR-1, FHR-1(SV), or FH18-20 were injected alone at 3, 7.5, or 50 µM, respectively (approximately 5-fold greater than the determined *K*_D_ of the respective analytes for biotinylated C3b immobilized onto the SPR chip; see **Supplementary Figure 1**), or in combination with FH at 1 µM (shown in red). **(B)** Human C3b opsonized RBCs that were either untreated or incubated with neuraminidase (desialylated) were exposed to FH (0.75 µM final concentration) or FH together with increasing FHR-1 or FHR-1(SV) concentrations. FH binding was detected with an anti-FH antibody and a secondary detection molecule with APC fluorescence marker. Data points are mean values (with SD) derived from at least three independent assays.

After addressing the question of why FHR-1 may protect foreign cell surfaces from complement activation, at least at high concentrations, insights into the mechanism of FH deregulation on host surfaces are still needed. We therefore employed an SPR experiment in which FH and either FHR-1 or FH18-20 are, first separately and then simultaneously, applied to immobilized C3b (**Figure 10A** and **Supplementary Figure 10A**). The sum of individual signals was not equal to that of FH and FHR-1 or FH18-20 injected simultaneously, indicating a competition for C3b binding. In contrast, when the experiment was repeated with FH and the variable domain of the complement receptor of the immunoglobulin family (CRIg), which binds C3b far away from the FH-binding site, the signals were indeed additive, thereby validating this assay (**Supplementary Figure 10B**). While confirming competition between FH and FHR-1 for C3b binding, the sensor chip assay does not provide a suitable explanation for why deregulation would occur on host but not on foreign cell surfaces. Significant deregulation can only be observed if FH does indeed bind to the cell surface, which is dependent on the presence of host patterns (sialic acid) and opsonization. Applying flow cytometry, we confirm that FH strongly binds to C3b-opsonized, sialylated human RBCs and that the removal of surface sialic acids results in a substantial reduction in FH recognition. This is in line with the expectation that FH adheres more strongly to host rather than to foreign or foreign-like (desialylated host) surfaces (**Figure 10B**). Importantly, in this host cell scenario, only the aHUS associated FHR-1(SV) variant showed a substantial deregulation activity at a 1:10 molar ratio, whereas wildtype FHR-1 could not considerably prevent binding of FH to C3b-opsonized human RBCs, even at a high molar excess of FHR-1 (**Figure 10B**).

## Discussion

The differential response of the complement system to self and foreign cell surfaces is largely mediated by the pattern recognition capacities of the FH protein family (3, 42). Despite valuable insights from molecular and disease-related studies, many aspects concerning the function of FHRs and their surface-dependent interplay with FH remain elusive. By analyzing structure-function relationships of the C-terminal constructs of FH and FHR-1 as well as full-length dimeric FHR-1, we set out to determine under which conditions FHR-1 activates or inhibits complement activation. To reveal the pathomechanism of an aHUS-associated FHR-1 variant, which contains two amino acid substitutions (L290S, A296V) that render the C-terminus identical to that of FH, we included constructs carrying these mutations in our characterization. For this we utilized several biophysical binding and serum-based complement assays. Heparin and C3b binding were not substantially affected when the C-terminal FH construct FH18-20(SV) was converted into the C-terminus of FHR-1*B, which corresponds to FH18-20(LA) (**Figure 1**). Wildtype FHR-1 and the FHR-1(SV) variant both showed consistently stronger binding to heparin and C3b when compared to all monomeric truncated FH variants, which underscores the impact of FHR-1’s dimeric nature. Strikingly, a slightly enhanced affinity for C3b could be observed for each construct carrying a leucine instead of serine (in position 290 for FHR-1 or in position 1,191 for FH18-20), which is consistent with a previous report of C-terminal mutant variants of FH CCP19-20 (43). In contrast, Heinen et al. demonstrated that FH18-20 (corresponding to S1191) binds with higher affinity to C3b than the three C-terminal domains of FHR-1 (corresponding to L290) (17). However, the overall affinity of the monomeric constructs for C3b was in the low µM range as in the present study. The S1191L substitution in FH18-20 substantially reduced the ability of FH18-20 to bind to α2,3-linked sialic acid, while the inverse substitution of L290S in FHR-1 converted FHR-1 into an enhanced sialic acid binder (**Figure 4** and **5**). The V1197A substitution in FH18-20 or A296V substitution in FHR-1 both had comparatively little impact on sialic acid binding. These experiments confirm earlier hypotheses that the difference between FHR-1 and FH in the C-terminal domains profoundly affects sialic acid binding and, consequently, host cell recognition (12, 26).

All constructs exhibiting binding to sialic acid substantially increased complement activity in human serum assays when self or self-like cell surfaces were tested (human endothelial cells, PNH erythrocytes, sheep erythrocytes, and guinea pig erythrocytes) (**Figures 6** and **7B**). This includes the FH18-20(SA) variant, wildtype FH18-20, and the aHUS associated FHR-1(SV) variant. An earlier study had already reported that plasma purified FHR-1(SV) strongly competes with FH for binding to cell surfaces impairing complement regulation by FH (26). Of note, at lower concentrations the dimeric FHR-1(SV) variant was substantially more efficient at increasing complement activation on host/host-like cells when compared to monomeric FH18-20. All non-(or weak) sialic acid binders showed only a comparatively small increase in complement activity at lower concentrations. However, especially on endothelial cells, increased complement activation can also be demonstrated for FHR-1 and FH18-20(LA) (**Figure 6A**). In contrast to host cells, on foreign (rabbit erythrocytes and yeast) or foreign-like cells (desialylated PNH or guinea pig erythrocytes) deregulation of FH by any of our C-terminal constructs was absent (**Figures 7** and **8**). Instead of increasing complement activation, the dimeric constructs even led to moderate protection of non-sialylated cell surfaces at high concentrations. Together these data indicate that deregulation of FH by FHR-1 occurs on host, rather than foreign surfaces. Although guinea pig erythrocytes lyse in human serum in the absence of any further addition of human FH, supplementation with human FH to 200 nM in 10% human serum is sufficient to protect guinea pig erythrocytes from AP-mediated hemolysis (22) indicating that moderate increases in human FH are sufficient for protection. This is in line with guinea pig erythrocytes being sialylated (35) and capable of expressing α2,3-linked sialic acid (36), which is the known binding partner for human FH (12, 13). Therefore, guinea pig erythrocytes, despite overall being activators of the human AP, recruit FH to their surface and thus are amenable for deregulation by FHR-1, as shown here (**Supplementary Figure 5**) and in previous studies (21, 22). Our experiments show a similar deregulation profile for different host cell surfaces. Further support for the notion that sialic acid moieties are important for FH deregulation comes from a study by Schmidt et al., which demonstrates that the ability of FH19-20 to increase lysis of human PNH-like cells is lost upon treatment of the RBCs with neuraminidase (31).

Active recruitment of FH to the cell surface is an essential prerequisite for competition or deregulation. When the recognition capacity of FH for C3b-opsonized human RBCs was assessed, as expected, FH only bound considerably to those cells under complement distress in which sialic acid moieties had not been removed (as detected by flow cytometry involving several washing steps; **Figure 10B**). This explains why not only conversion of the C-terminus of FHR-1 to the C-terminus of FH is pathogenic but also the inverse. Several publications have described the occurrence of aHUS in individuals with FH molecules that resembled the C-terminus of FHR-1 generated by gene conversion or non-allelic homologous recombination (44, 45). On desialylated and C3b-opsonized RBCs, only low levels of FH binding could be detected by flow cytometry. Contrary to expectations, neither FHR-1 nor the aHUS-associated FHR-1(SV) variant could compete FH off the C3b-opsonized, sialic acid-bearing RBCs at physiological ratios of FH and FHR-1. Notable deregulation could only be detected when a substantial molar excess of FHR-1(SV) over FH was applied. One potential explanation for this observation could be that the high density of C3b molecules on the *in vitro* opsonized human RBCs outnumbers a majority of FH and FHR-1 molecules in the assay and thus, competition cannot effectively take place. Utilizing C3b-coated SPR sensor chips, which are particularly well suited to detect weak interactions in a dynamic setting, a direct competition of FH and FHR-1 (or FHR-1(SV)) for deposited C3b was evident (**Figure 10A**). In all serum assays on host and host-like cells the sialic acid binders FHR-1(SV) and FH18-20 strongly increased complement activation, indicating competition with FH on host surfaces. This demonstrates that the aHUS-associated mutations in the FHR-1(SV) C-terminus, which becomes identical to FH, converts FHR-1 into a sialic binder that more efficiently competes with FH. This enhanced deregulation capacity on host cells bearing α2,3-linked sialic acid, including renal endothelial cells, may largely explain the disease association of the SV variant. Interestingly, a slight yet notable increase in complement activity for wildtype FHR-1 was only observed on endothelial cells (**Figure 6A** insert) but not RBCs. Even under normal conditions, low constitutive binding of FHR-1 to renal endothelial cells, which also contain a specific subset of GAGs, may render this cell type more susceptible to deregulation. The profoundly increased complement activation potential of FHR-1(SV) in the HMEC-1 assay provides a strong rationale for why increased deregulation by the FHR-1 variant leads to a loss of protection and strong activation of the AP and terminal pathway that defines aHUS.

FHR-1 was previously reported to negatively regulate C5 convertases but not C3 convertases (17). Heinen et al. employed an assay in which convertases were assembled on sheep erythrocytes in human serum depleted of FH and FHR-1, and activation of C3 and C5 was measured upon adding increasing amounts of FHR-1. In this setting, FHR-1 could reduce the activation of C5, while C3 conversion was not affected. In line with these results, we show that rabbit RBCs as a model of non-self cells were substantially protected from lysis by high concentrations of FHR-1 or FHR-1(SV) (**Figure 7C**). We show that FHR-1 competes with C5 for binding to C3b explaining the underlying mechanisms of how FHR-1 acts on the C5 convertase (**Supplementary Figure 9**). Previously, Zwarthoff *et al.* have already shown that FHR-5 can inhibit the access of C5, but not of C3, to surface bound convertases (46). We also failed to observe an interference by FHR-1 for C3 binding to C3b (**Supplementary Figure 8**). However, our SPR-based assay for direct C3b binding and the reported surface-based cell assays with preassembled convertases (17, 46) did not consider whether FHR-1 influences *in situ* convertase assembly. In another SPR-based assay we show that this, indeed, is the case. In the presence of the dimeric constructs FHR-1 and FHR-1(SV), fewer convertases are assembled when FB and FD are applied to deposited C3b (**Figure 9**). Again, this effect was observed when very high concentrations of FHR-1 (about tenfold over the *K*_D_ for C3b; i.e., 9.6 µM of dimeric FHR-1 equating to 680 µg/ml) were applied. Previously, a plate-based assay using much lower FHR-1 concentrations (50 µg/ml) failed to detect any interference with formation of the AP C3 convertase (47). Direct inhibitory effects of FHR-1 on the complement cascade are therefore likely limited to conditions involving supra physiologically high concentrations of FHR-1. Similar to the effect on the C5 convertase this phenomenon only occurs for the dimeric full-length constructs of FHR-1 and is absent from the monomeric C-terminal fragments spanning three CCP domains. It is therefore reasonable to assume that the size/volume of dimeric constructs sterically hinder the access of C5 or FB to their binding partner C3b. While C3 and C5 are believed to share a common binding area on MG4-7 of C3b, an additional patch on C3b for C5 binding had been hypothesized previously (39, 41, 48). For the interaction of FB the C-terminal domain of C3b is critical (49). This may well explain why C3b competition in the presence of FHR-1 is only observed for FB and C5 but not for C3.

**Figure 11:**
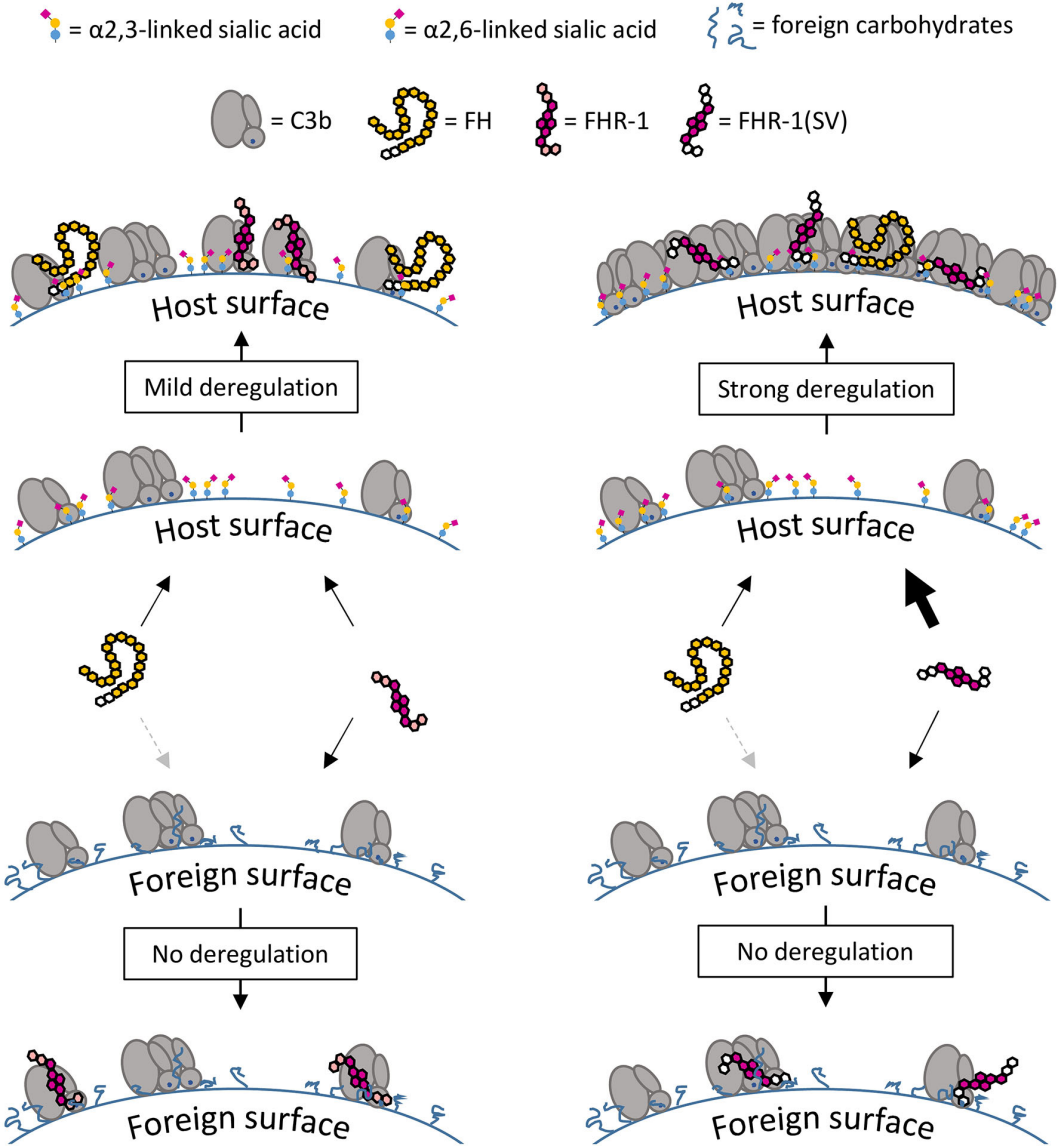
Surface-dependent deregulation of FH. On C3b opsonized host surfaces FH interacts with C3b and α2,3-linked sialic acid, whereas FHR-1 interacts with C3b only, but the dimeric structure of FHR-1 enhances competition with increasing C3b deposition. The inability of FHR-1 to bind α2,3-linked sialic acid acts as a safety mechanism that prevents strong competition with FH on host surfaces. Strong deregulation and subsequent opsonization are a result of the amino acid exchanges in FHR-1(SV) which creates a dimeric molecule that binds to C3b and α2,3-linked sialic acid. On foreign surfaces there is almost no competition and therefore no deregulation is observed; FH binds weakly to these surfaces due to the lack of the second binding partner α2,3-linked sialic acid.

The C3b opsonization assay on yeast demonstrated that only high concentrations of FHR-1 and FHR-1(SV) were able to substantially reduce the C3 density on these cells and thus considerably inhibit the formation of C3 convertases (**Figure 8**). Such protective effects of the dimeric constructs materialize only on foreign cells, on which FH is not sufficiently bound and thus cannot be competed, i.e., deregulated. On human endothelial cells, C3b opsonization becomes more pronounced with increasing concentrations of FHR-1 and FHR-1(SV) (**Figure 6A**), which impressively highlights the context-dependent role of FHR-1. Context dependent roles of FHR-1 have also been described in other reports for apoptotic and necrotic cells [reviewed in (50, 51)]. Whether the observed inhibitory effect toward the C3 and the C5 convertases plays an important physiological role *in vivo* must be questioned for two reasons. Firstly, the convertase inhibitory effect is only seen on foreign surfaces at very high, supraphysiological concentrations. Secondly, the convertase inhibitory effect was not observed on human host surfaces, which suggests that the competition with FH prevails on these cells and, on the whole, leads to more complement activation (**Figure 11**).

In conclusion, our studies provide critical insights into the molecular and functional determinants of complement modulation by FHR-1. While we confirm that FHR-1 may inhibit the C5 convertase by restricting access of C5 to the convertase, and uncover that FHR-1 can impair the assembly of the AP C3 convertase, these effects were only observed at high concentrations and render the physiological relevance uncertain. Nevertheless, these insights will be valuable in light of the contradictory views in the literature. More importantly, however, we uncover that FHR-1 deregulates FH on host but not foreign cells, and provide a molecular rationale for the disease mechanisms of the aHUS-associated FHR-1(SV) variant. The amino acid substitutions L290S and, to a minor degree, A296V in this variant convert FHR-1 into a sialic acid-binding protein with substantially pronounced deregulation capacity on host cell surfaces under complement distress, as observed in aHUS and other disorders.

## Data Availability Statement

The original contributions presented in the study are included in the article/**Supplementary Material**. Further inquiries can be directed to the corresponding author.

## Ethics Statement

The studies involving human participants were reviewed and approved by Ethical Committee of Ulm University (Ethikkommission der Universität Ulm). The patients/participants provided their written informed consent to participate in this study.

## Author Contributions

All authors contributed to the article and approved the submitted version.

## Funding

This work was supported by a grant from Deutsche Forschungsgemeinschaft (SCHM3018/2-2 to CS) and the Swiss National Science Foundation (31003A_176104 to DR).

## Conflict of Interest

CS, BH, and HS are inventors of a patent application that describes the use of engineered complement regulatory proteins for therapeutic applications. CS and BH have received honoraria for speaking at symposia organized by Alexion Pharmaceuticals. BH acted as a consultant for various pharmaceutical companies. HS received research grants and honoraria for speaking in symposia or service on advisory boards from Alexion Pharmaceuticals, Roche, Apellis, and Ra Pharmaceuticals (all to University Hospital Ulm). DR is an inventor of patents and patent applications describing complement inhibitors and their clinical use. He received honoraria for speaker and consulting engagements from various pharmaceutical companies.

The remaining authors declare that the research was conducted in the absence of any commercial or financial relationships that could be construed as a potential conflict of interest. 
