# Supplementary material for: Deregulation of Factor H by Factor H-Related Protein 1 Depends on Sialylation of Host Surfaces
Source: Front Immunol. 2021 Feb 25;12:615748. doi: 10.3389/fimmu.2021.615748 (PMC7959842; doi:10.3389/fimmu.2021.615748)
Supplement: Supplementary file 1 [file DataSheet_1.pdf]

SUPPLEMENTAL MATERIAL online

Deregulation of Factor H (FH) by Factor H-related protein 1 (FHR-1) depends on sialylation of host surfaces

Arthur Dopler[1], Selina Stibitzky[1], Rachel Hevey[2], Marco Mannes[1], Mara Guariento[1+], Britta Höchsmann[3], Hubert Schrezenmeier[3], Daniel Ricklin[2] and Christoph Q. Schmidt[1*]

[1]Institute of Pharmacology of Natural Products and Clinical Pharmacology, Ulm University, Ulm, Germany

[2]Department of Pharmaceutical Sciences, University of Basel, Basel, Switzerland

[3]Institute of Transfusion Medicine, University of Ulm, Ulm, Germany and Institute of Clinical Transfusion Medicine and Immunogenetics Ulm, German Red Cross Blood Transfusion Service and University Hospital of Ulm, Ulm, Germany;

[+]present address: Rentschler Biopharma SE, Erwin-Rentschler-Str. 21, 88471 Laupheim, Germany

*** Correspondence:**
Christoph Q. Schmidt,

christoph.schmidt@uni-ulm.de

1) Supplemental Figures (page 2-18)
2) Supplemental Methods (page 19)

Supplemental Fig. 1

Binding to biotin coupled C3b

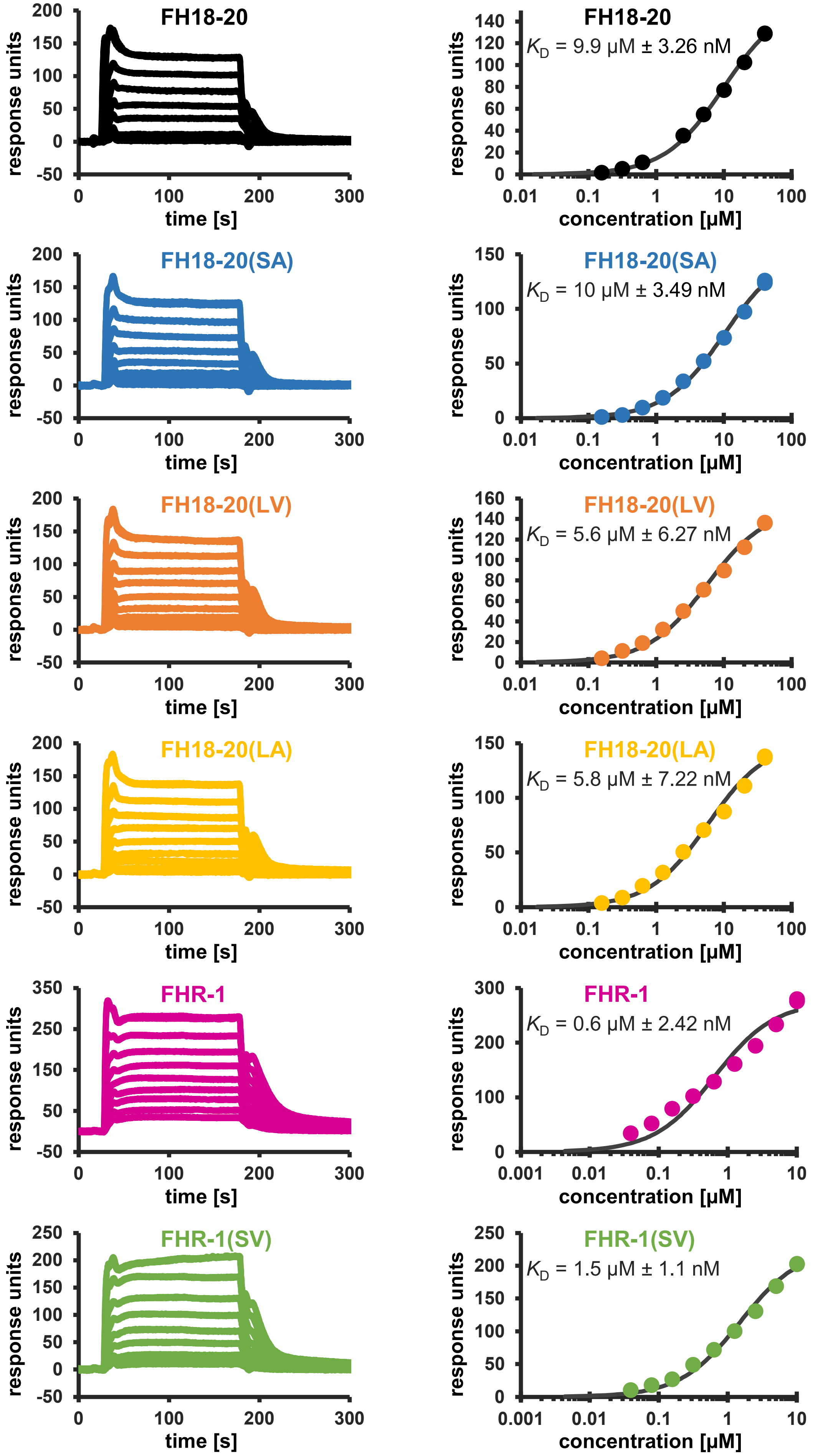

Supplemental Fig. 1: Binding affinity to biotin-coupled C3b assessed by SPR. SPR sensorgrams (not normalized to molecular weight) of C3b binding and corresponding response–concentration plots with 1:1 steady-state affinity fits are shown. Recombinant FH18-20 or FHR-1 constructs were flowed over a streptavidin (SAP) biosensor chip with 1500 RUs of biotin-coupled C3b (left panels). The corresponding concentration–response plots with the extracted K_D values are shown in the right Panels. Only reference-subtracted sensorgrams are shown.

Supplemental Fig. 2

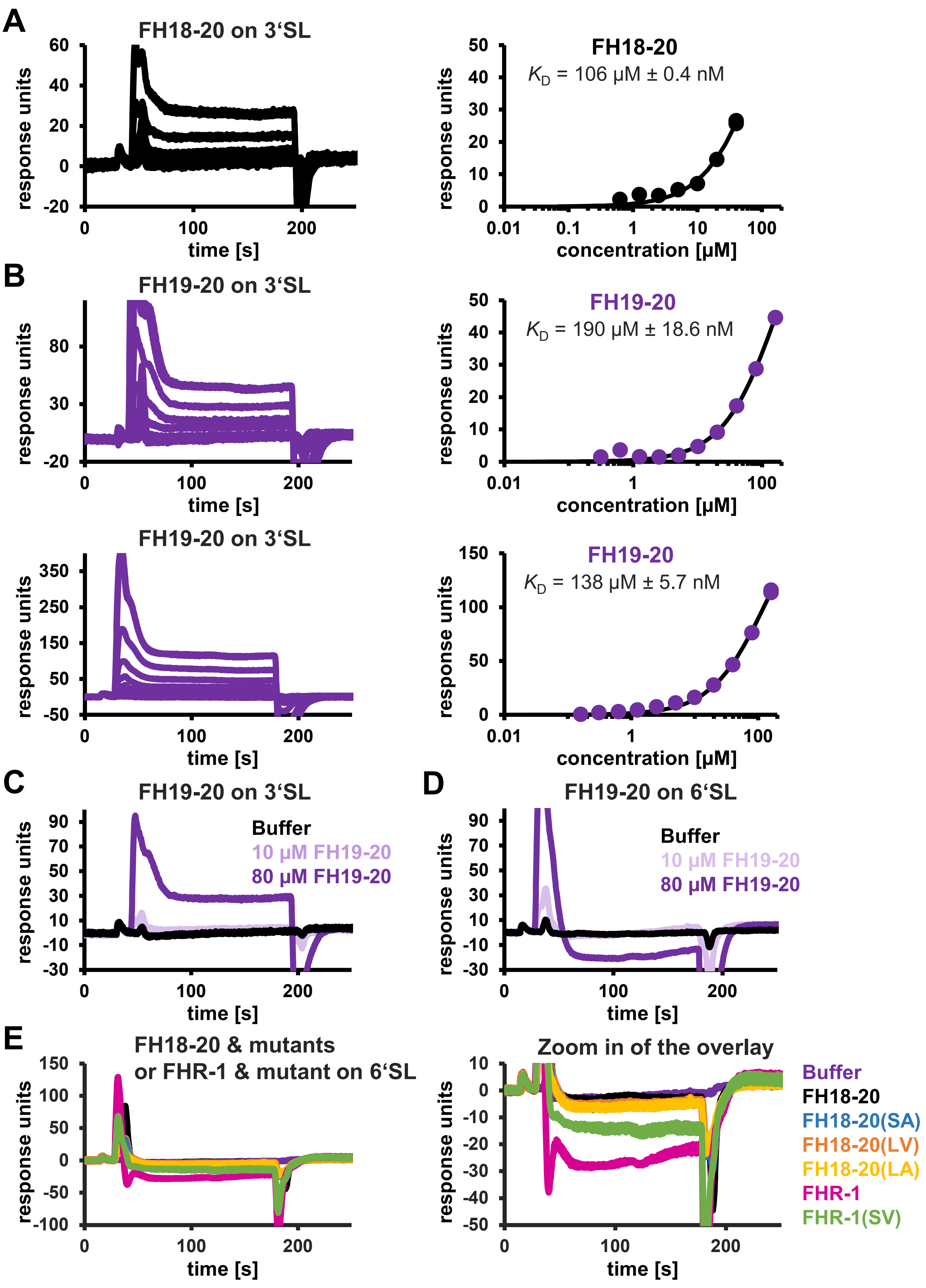

Supplemental Fig. 2: Sialic acid affinity measurements by SPR. The affinity of FH18-20, FHR-1 and their mutant versions for sialic acid was measured by SPR. All proteins were flowed over a streptavidin sensor chip immobilized with either α2,6-linked (6'SL) or α2,3-linked sialic acid (3'SL). Only reference-subtracted sensorgrams are shown. **A.** FH18-20 was injected from 40 to 0.63 µM (1 in 2 dilution series) on a chip with approximately 150 RU of immobilized α2,3-linked sialic acid. The corresponding response–concentration plots with 1:1 steady-state affinity fits and K_D values are shown in the right panel **B.** Same as in A but with FH19-20 from 160 to 0.31 µM on two different chips (approximately 165 RU α2,3-linked sialic acid in the upper panels and approximately 240 RU in the lower panels). Sensorgrams of FH19-20 at 10 or 80 µM injected on a chip immobilized with approximately **C.** 165 RU α2,3-linked sialic acid or **D.** 150 RU α2,6-linked sialic acid. **E.** Sensorgrams of the FH18-20 and FHR-1 constructs injected on a chip immobilized with approximately 150 RU α2,6-linked sialic acid. The right panel shows an enlargement of the overlay for better comparison.

Supplemental Fig. 3

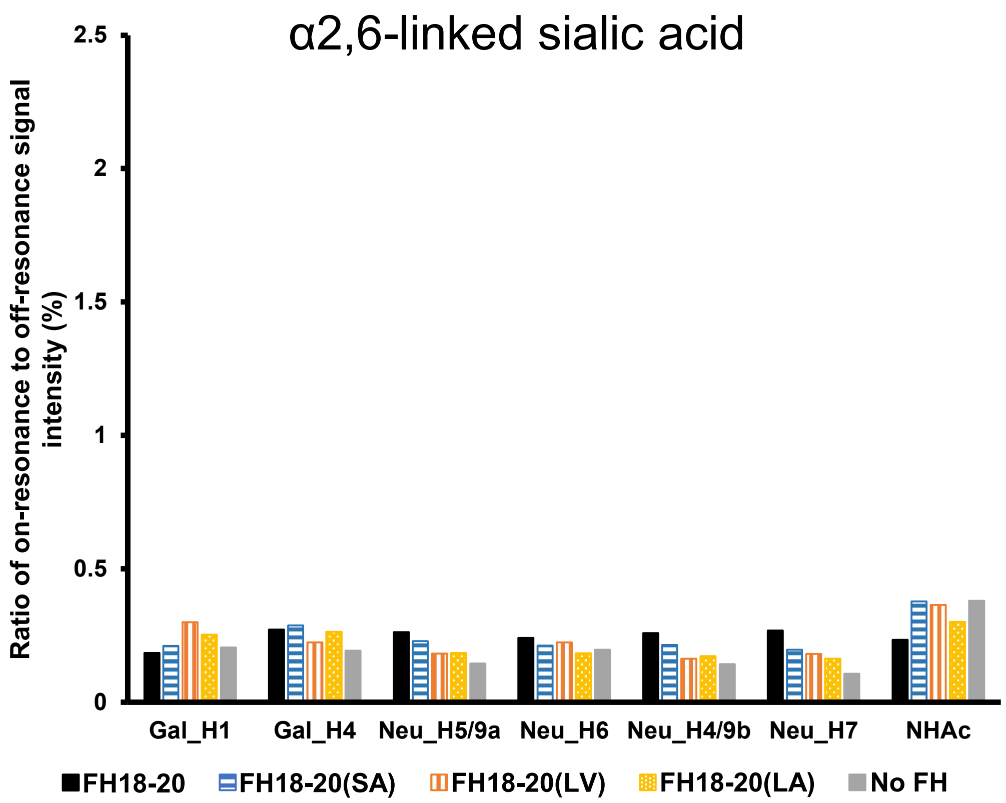

Supplemental Fig. 3: α2,6-linked sialic acid affinity measurements by STD-NMR. α2,6-linked sialic acid binding of FH18-20 and its mutational variants was determined by STD-NMR. The histograms report the ratio of on-resonance to off-resonance signal intensities (%) received upon binding of FH18-20 constructs to different hydrogen atoms (H) of α2,6-linked sialic acid.

Supplemental Fig. 4 (1)

HMEC1 assay 1

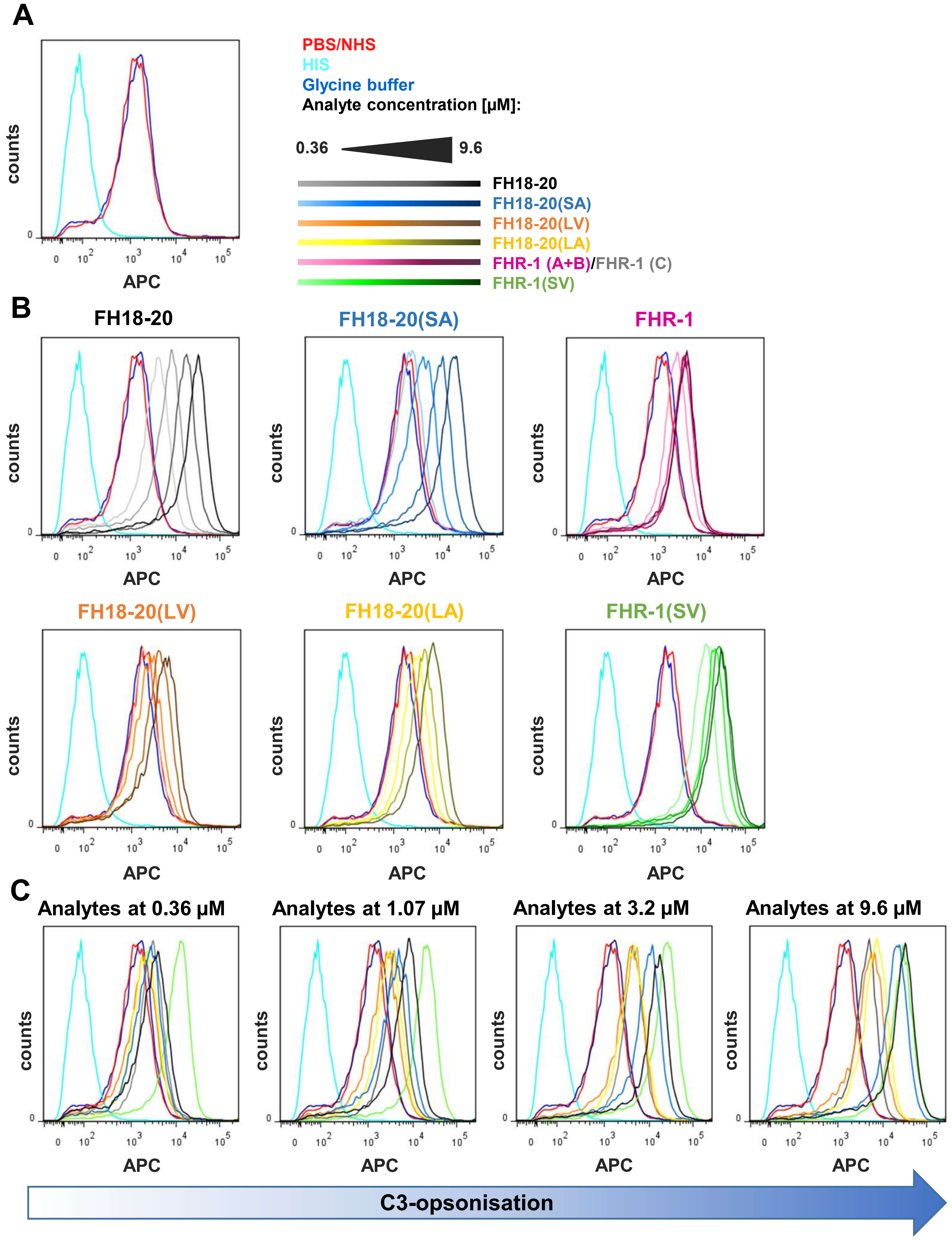

Supplemental Fig. 4 (2)

HMEC1 assay 2

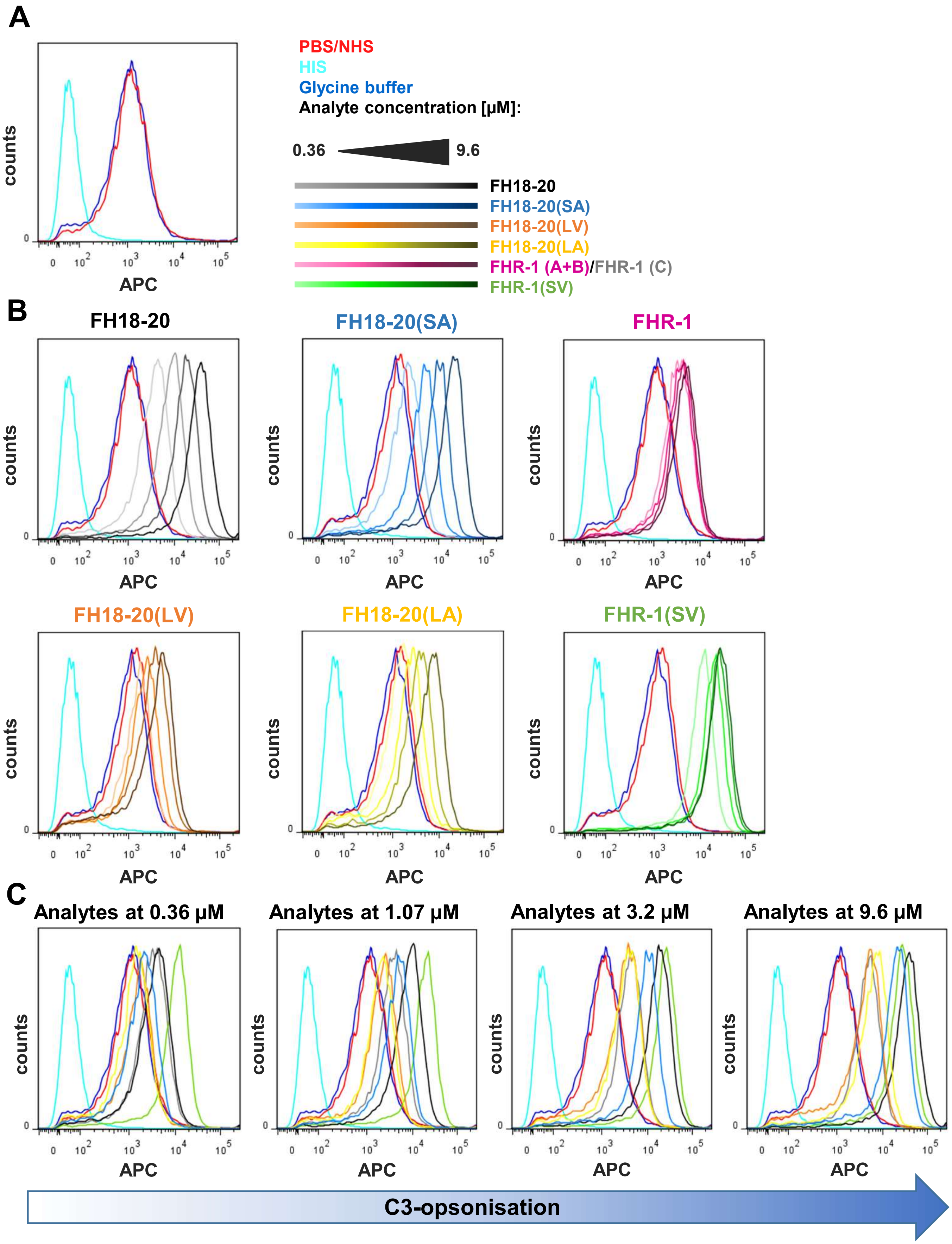

Supplemental Fig. 4 (3)

HMEC1 assay 3

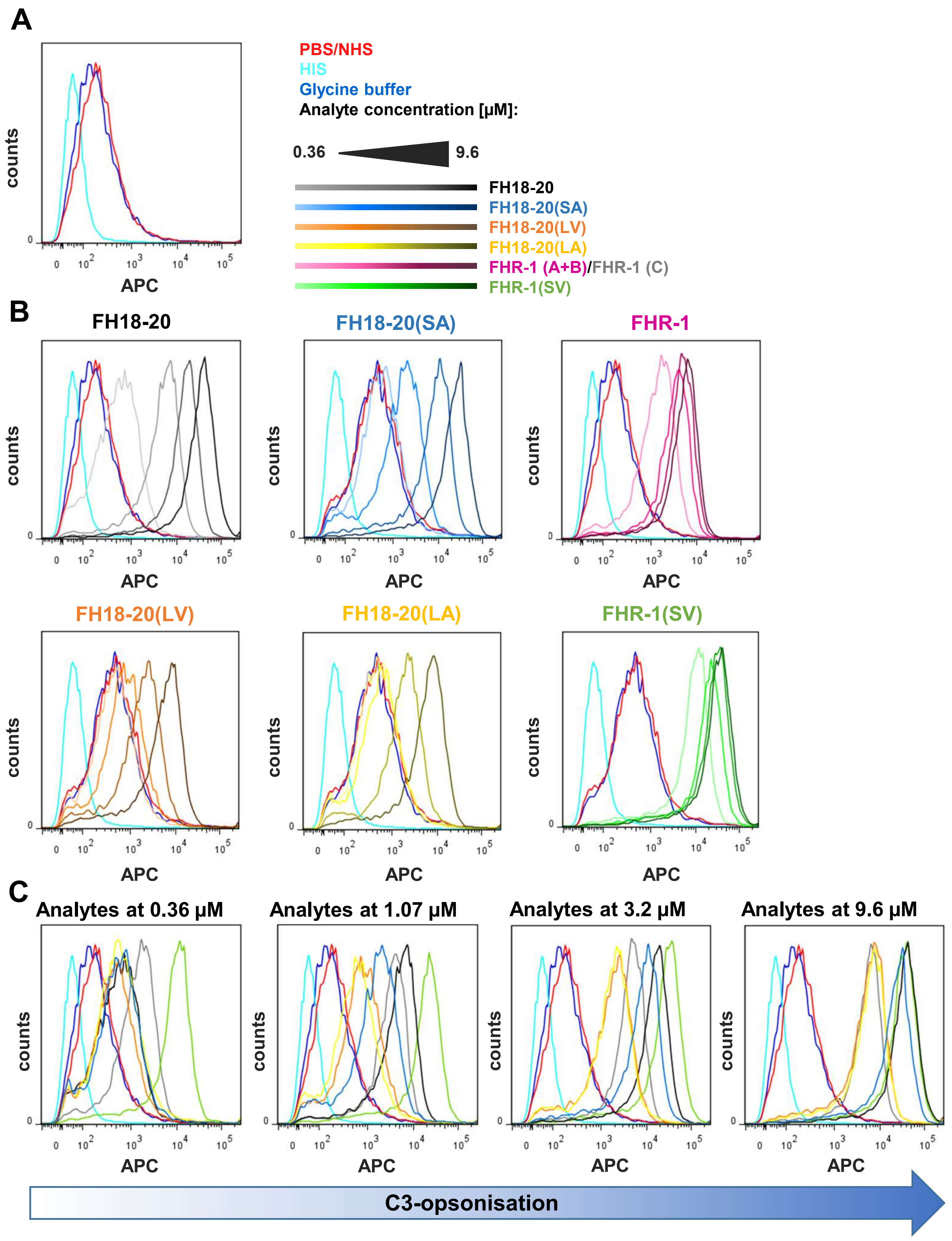

Supplemental Fig. 4: Adherent human microvascular endothelial cells (HMEC-1) opsonization assay. Flow cytometric analysis of HMEC-1 cells after exposure to serum mixed with increasing amounts (0.36-9.6 µM) of the different FH18-20 and FHR-1 analytes. Cells were labelled with anti-C3d antibody and a secondary detection molecule with APC fluorescence marker to measure levels of C3-opsonisation. As controls NHS (red) and HIS (cyan), each mixed with PBS as analyte, and NHS mixed with the highest concentration of glycine buffer used in the sample containing 9.6 µM of FHR-1(SV) were used. **A.** The controls without protein analytes are shown. **B.** The histograms of each analyte series and of the controls are shown. In each series the increasing concentration of the analyte is indicated by a corresponding colour gradient. **C.** Overlay of the histograms of all proteins at all used concentrations together with the controls are shown. FH18-20, FH18-20(SA), FH18-20(LV), FH18-20(LA), FHR-1 and FHR-1 (SV) are shown in black, blue, orange, yellow, grey and green, respectively. FlowJo (version 7.6.5) was used as evaluation tool. Each figure shows one of three independent experiments.

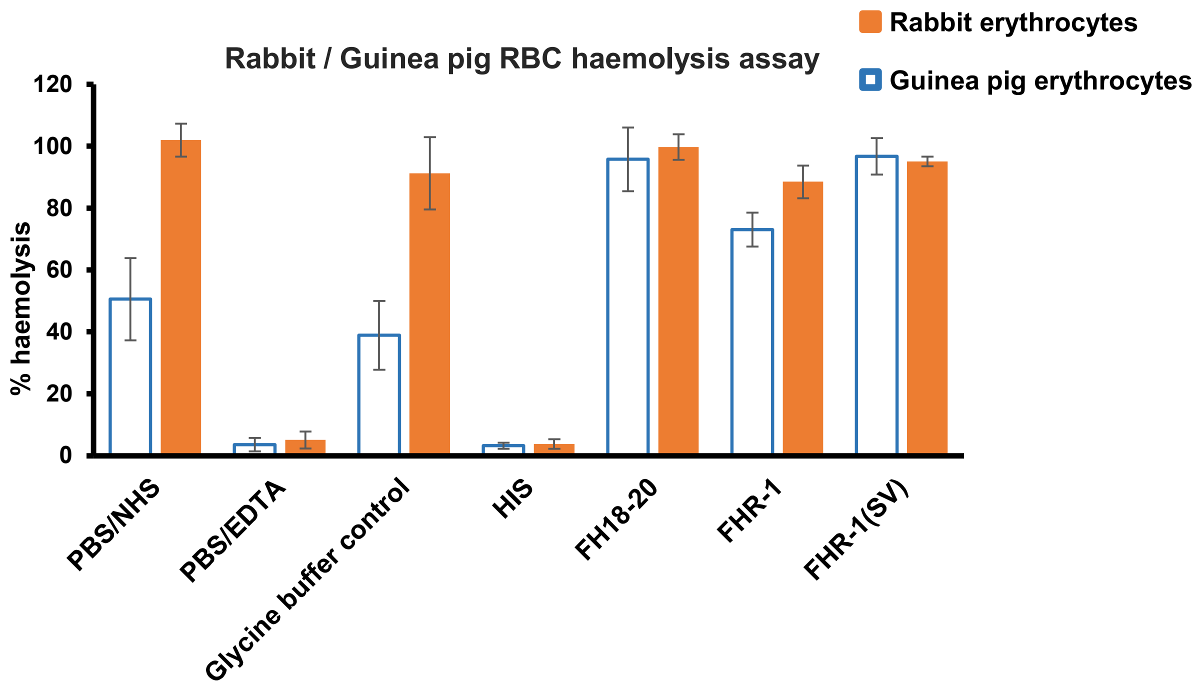

Supplemental Fig. 5: Functional cell assay on foreign and self-like surfaces. Guinea pig (white with blue border) or rabbit (orange) erythrocytes were incubated with NHS (40 % final serum percentage) in presence of 9.6 µM FH18-20, FHR-1 or FHR-1(SV). Lysis and subsequent release of haemoglobin was determined by UV absorbance at 405 nm. All values were normalised to lysis in water (100% lysis mark). The mean percentage of haemolysis of three independent assays, each conducted in duplicate, is shown with SD. As controls NHS and HIS, each mixed with PBS instead of a protein analyte, were used. As a further control NHS was mixed with the highest concentration of glycine buffer used in the assay. A fourth control was added, which consisted of NHS mixed with PBS containing EDTA (5 mM final concentration).

Supplemental Fig. 6

Yeast opsonization assay 2

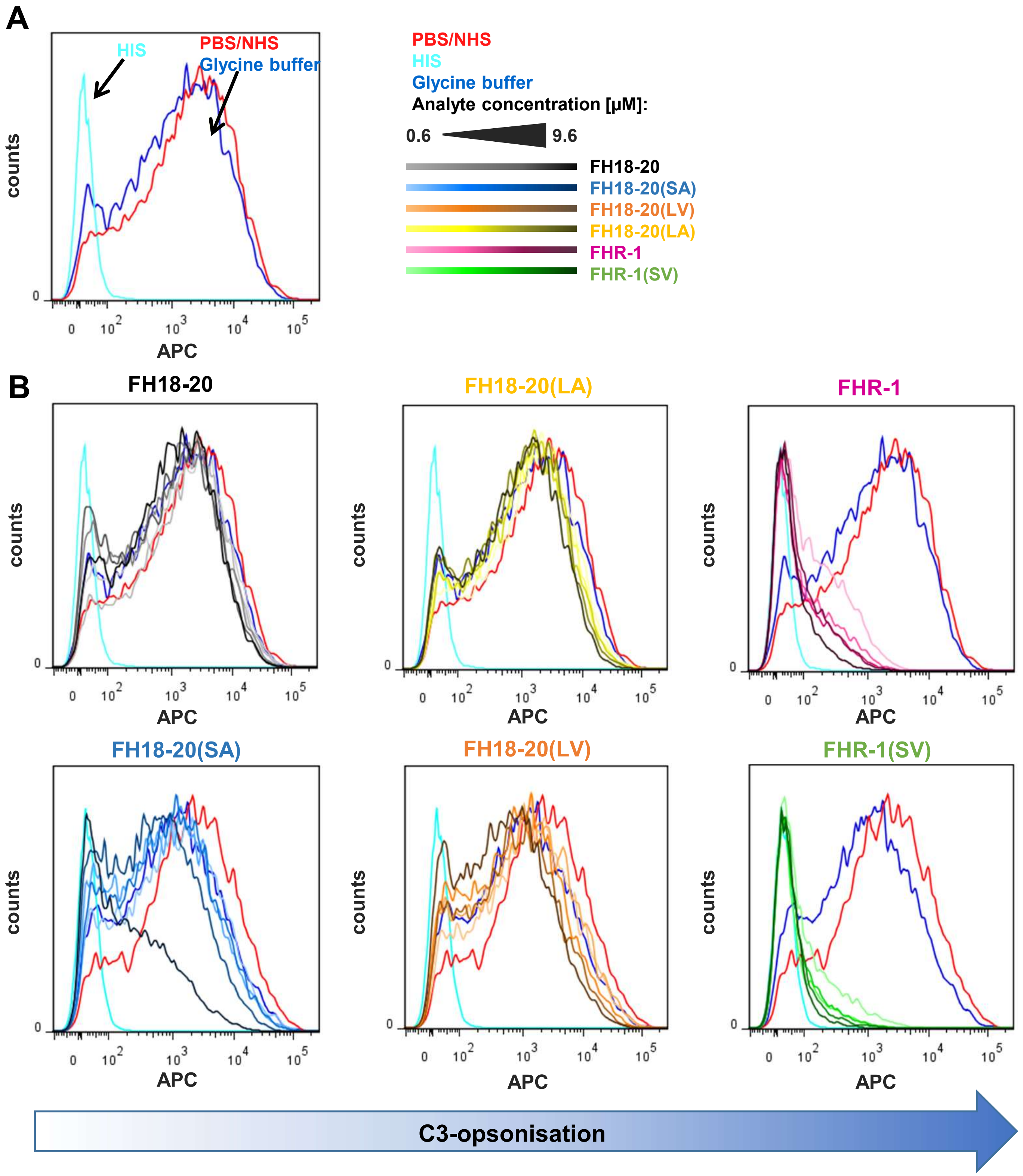

Supplemental Fig. 6: Yeast opsonization assay. Flow cytometric analysis of *P. pastoris* KM71H yeast cells after exposure to serum mixed with increasing amounts (0.6-9.6 µM) of the different FH18-20 and FHR-1 analytes. Cells were labelled with anti-C3d antibody and a secondary detection molecule with APC fluorescence marker to measure levels of C3-opsonisation. As controls NHS (red) and HIS (cyan), each mixed with PBS as analyte, and NHS mixed with the highest concentration of glycine buffer used in the sample containing 9.6 µM of FHR-1(SV) were used. **A.** The controls without protein analytes are shown. **B.** The histograms of each analyte series and of the controls are shown. In each series the increasing concentration of the analyte is indicated by a corresponding colour gradient. One of two independent experiments is shown. FlowJo (version 7.6.5) was used as evaluation tool.

Supplemental Fig. 7

Inhibition of AP C3 convertase formation

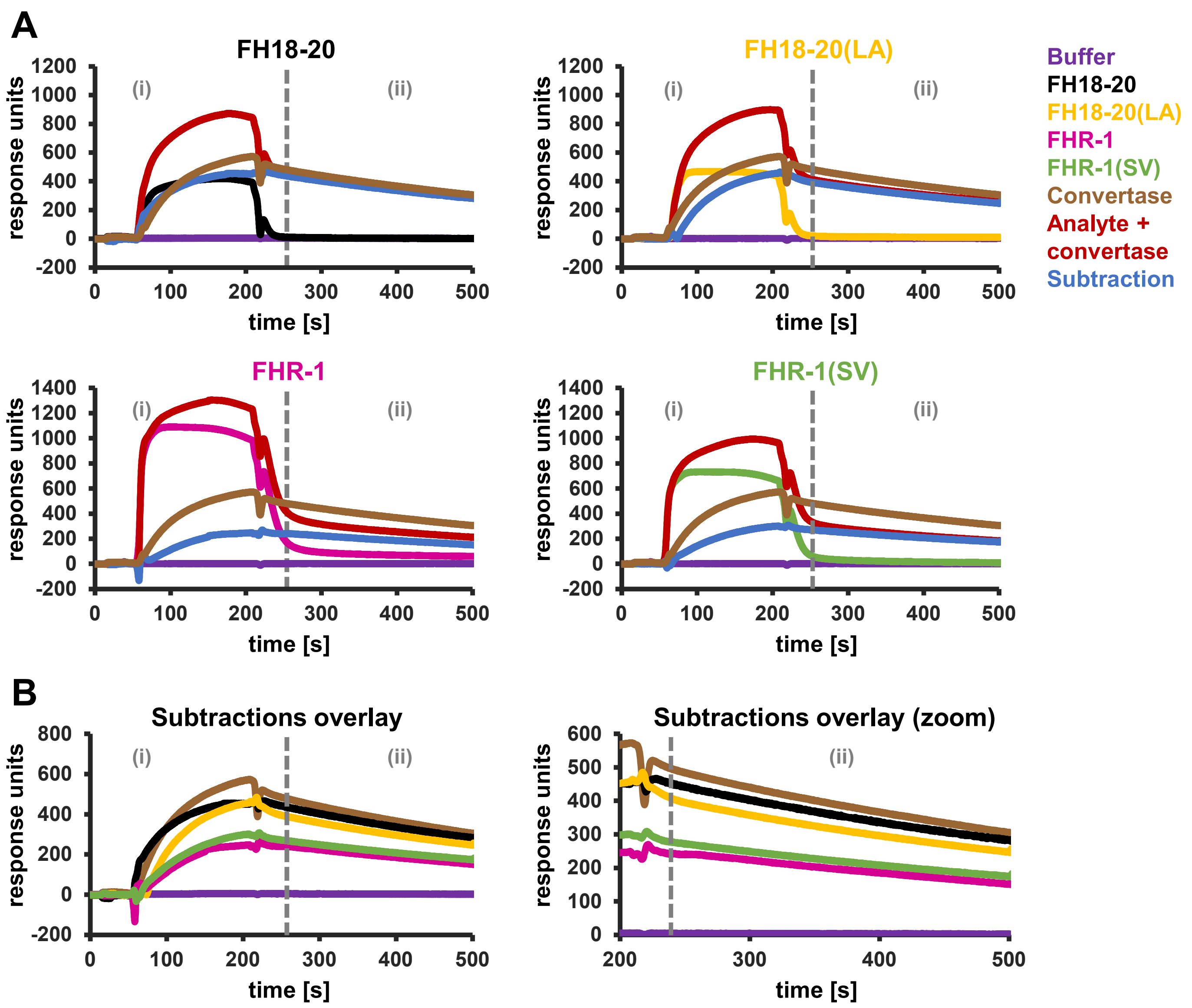

Supplemental Fig. 7: Inhibition of AP C3 convertase formation. A. Effect of FH18-20, FHR-1 and their respective double mutants on AP C3 convertase formation. In each panel four different sensorgrams are shown, corresponding to different protein mixtures: the signal of the AP C3 convertase alone, obtained by injection of FB (600 nM) and FD (100 nM) over a CMD500m sensor chip with 6000 RUs of amine-coupled C3b immobilized, is shown in brown. The signal of the analyte alone, FH18-20, FH18-20(LA), FHR-1 or FHR-1 (SV) (injected at a concentration ten times higher than the measured K_D value), is shown in black, yellow, pink and green, respectively. In dark red the signal obtained by injecting one of the analyte together with FB and FD (analyte + convertase) is shown. The blue sensorgram corresponds to the curve obtained subtracting the signal of the analyte alone from that of the analyte + convertase (subtracted sensorgrams). Buffer signal is shown in purple. **B.** Overlay of all subtracted sensorgrams (as seen in **A.**) with the signal of the convertase alone (brown). (i) Analytes binding to C3b is shown. (ii) Natural decay of the formed C3 convertase is shown. All shown sensorgrams are reference-subtracted.

Supplemental Fig. 8

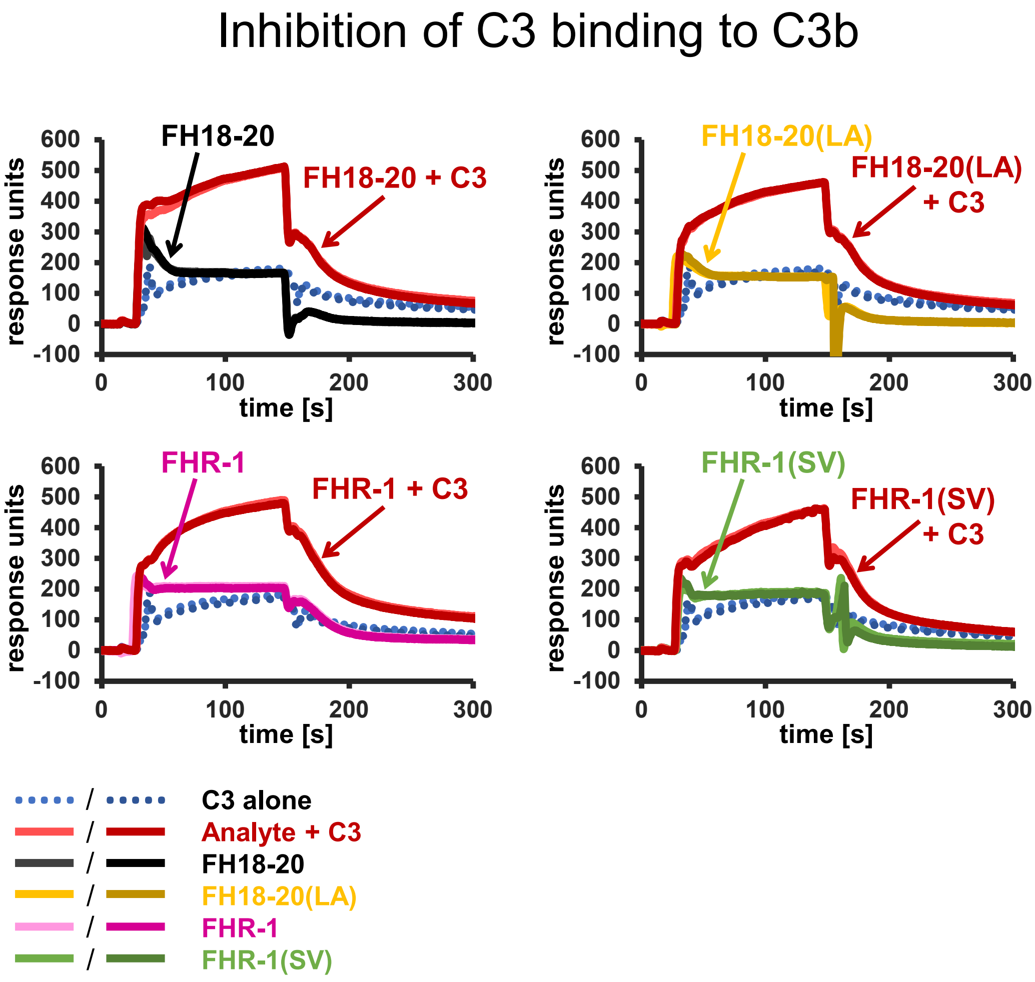

Supplemental Fig. 8: Inhibition of C3 binding to C3b. Effect of FH18-20, FHR-1 and their double mutants on C3 binding to C3b. In each panel three different sensorgrams are shown: the signal of C3 alone (1 µM) binding to a streptavidin sensor chip with 2300 RUs of immobilized biotinylated C3b is shown as a dotted blue line. The curves corresponding to the signal of the analyte alone (injected at a concentration ten times higher than the measured KD value) are shown in solid black, yellow, pink and green (two shades of each colour to indicate duplicates) for FH18-20, FH18-20(LA), FHR-1 or FHR-1 (SV), respectively. In red and dark red are shown the signals, in duplicate, corresponding to the binding of the analyte/C3 mixture to C3b. All shown sensorgrams are reference-subtracted.

Supplemental Fig. 9

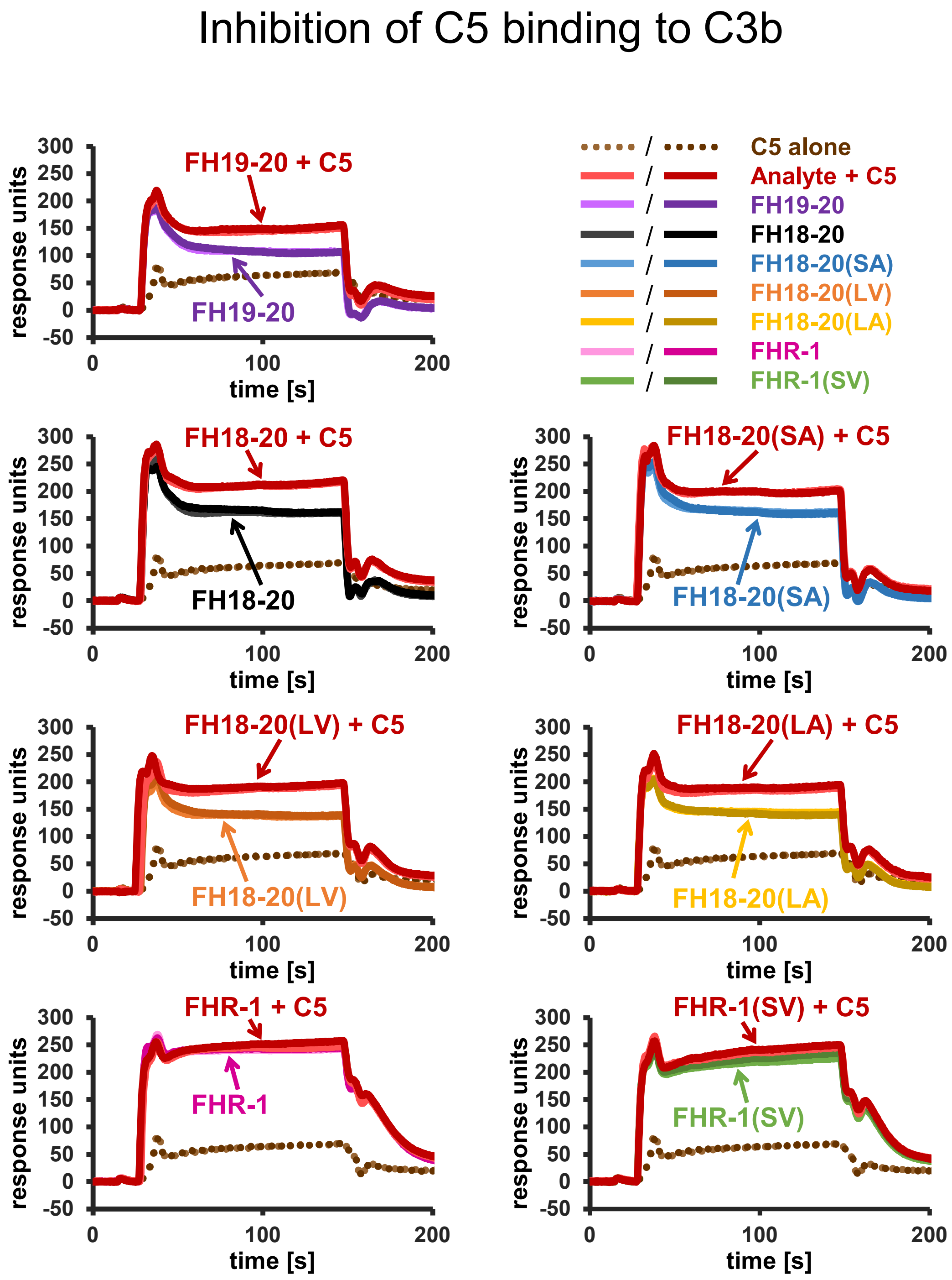

Supplemental Fig. 9: Inhibition of C5 binding to C3b. Effect of FH19-20, FH18-20, FHR-1 and their mutants on C5 binding to C3b. In each panel three different sensorgrams are shown: the signal of C5 alone (0.15 µM) binding to a streptavidin sensor chip with 1500 RUs of immobilized biotinylated C3b is shown as a dotted brown line. The curves corresponding to the signal of the analyte alone (injected at a concentration ten times higher than the measured KD value) are shown in solid purple, black, blue, orange, yellow, pink and green (two shades of each colour to indicate duplicates) for FH19-20, FH18-20, FH18-20(SA), FH18-20(LV), FH18-20(LA), FHR-1 or FHR-1 (SV), respectively. In red and dark red are shown the signals, in duplicate, corresponding to the binding of the analyte/C5 mixture to C3b. All shown sensorgrams are reference-subtracted.

Supplemental Fig. 10

Competition on C3b

A

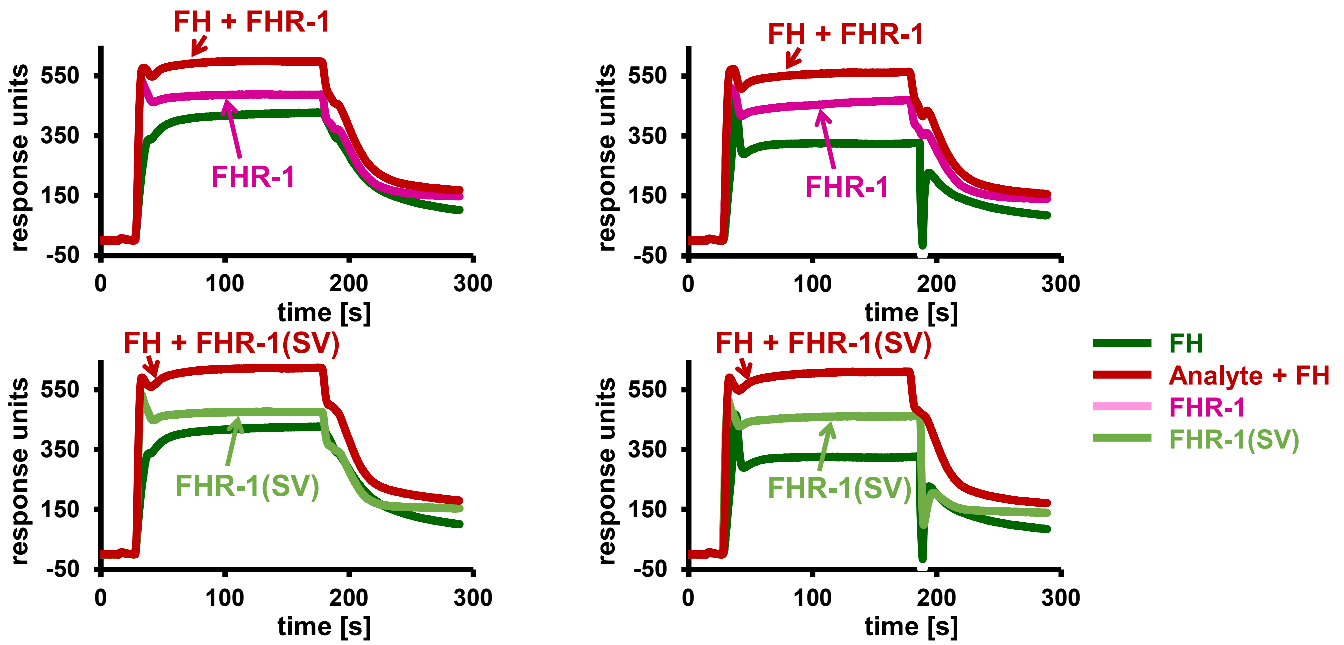

Addition on C3b

B

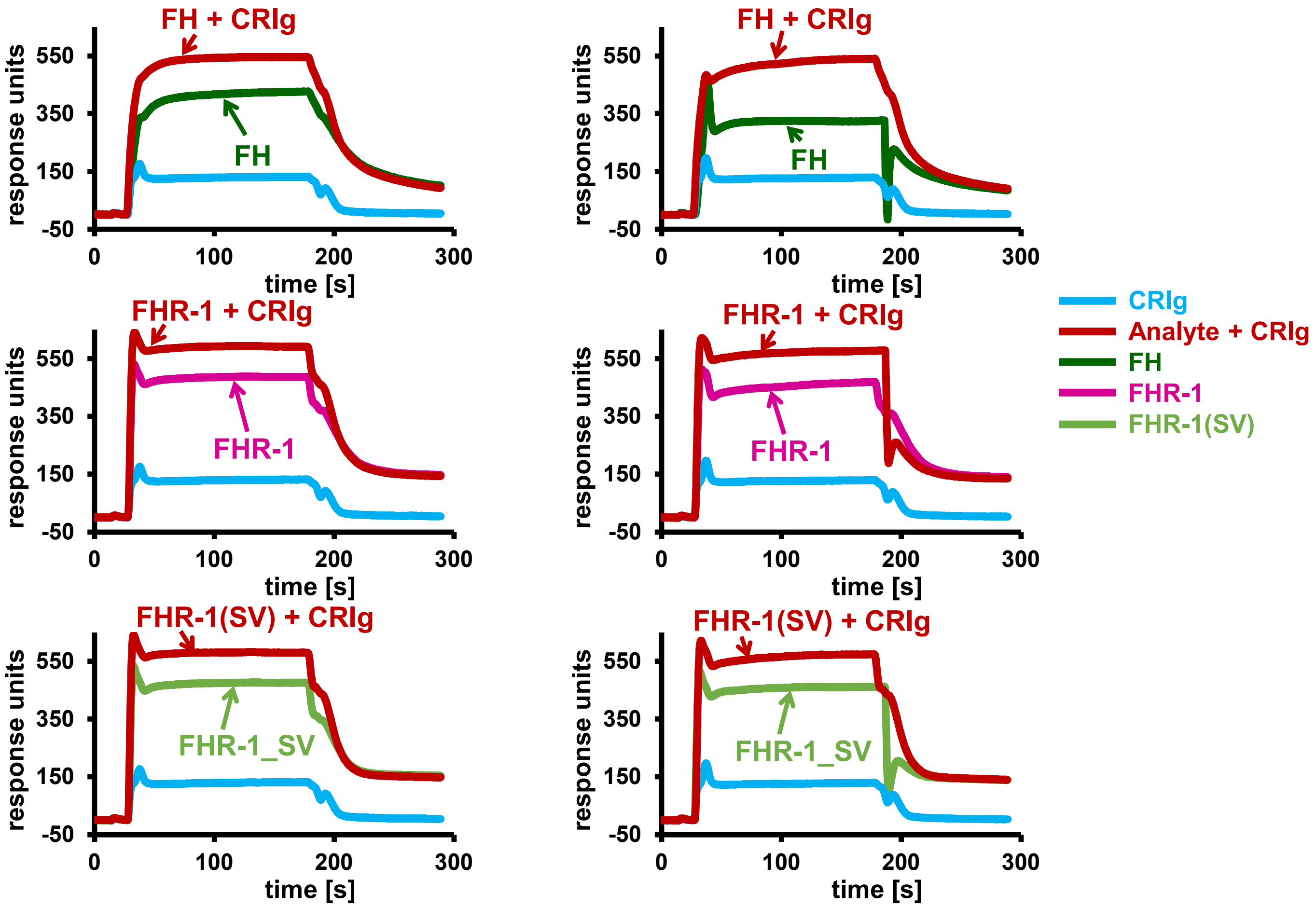

Supplemental Fig. 10: Competition of FH with FHR-1 constructs on C3b measured by SPR. Approximately 2500 RUs biotinylated C3b was immobilized on a Streptavidin chip and FHR-1 and the mutant variant FHR-1(SV) was injected either alone or in combination with FH to allow competition for C3b binding. CRIg, which is known to bind another site than FHR-1/FH, was injected in combination with FH, FHR-1 or FHR-1(SV) as a control. All injections were performed twice with the first injection shown on the left side and the second on the right. Only reference subtracted sensorgrams are shown.

Supplemental Methods

Inhibition of AP C3 convertase formation

The inhibition of AP C3 convertase formation was tested by SPR. Approximately 6000 RUs of C3b were immobilised by standard amine coupling on a CMD500M chip (XanTec Bioanalytics), which had been previously conditioned and washed according to manufacturer's recommendations. First the AP C3 convertase signal alone was determined injecting a mixture of FB (600 nM) and FD (100 nM) for 3 min and observing the natural C3 convertase decay for 5 min. In each separate experiment the analyte alone (FH18-20, FH18-20(LA), FHR-1 or FHR-1(SV), all 9.6 µM) or a mixture of the analyte (9.6 µM), FB (600 nM) and FD (100 nM) was injected for 3 min and the natural C3 convertase decay was observed for 5 min. After every injection, 1 µM recombinant CR1 CCP1-3 was flowed over the chip for 0.5 min to remove the remaining convertases by accelerating the decay. For regeneration, 1 M NaCl was injected for 0.5 min. All experiments were performed at 25 °C in PBS containing 0.005% Tween20 and 1 mM $MgCl_2$ at a flow rate of 25 µl/min. Finally, the TraceDrawer software was used to subtract the C3b binding signal of the analyte alone from the signal obtained for the analyte/convertase mixture.

Inhibition of C3 binding to C3b

SPR was used to test if FH18-20, FH18-20(LA), FHR-1 or FHR-1(SV) inhibit C3 binding to C3b. Approximately 2300 RUs of biotinylated C3b (prepared as previously described) were immobilised on a streptavidin chip (SAP; XanTec Bioanalytics), previously conditioned and washed according to manufacturer's recommendations. First, the signal of C3 binding to C3b was measured by injecting 1 µM C3 for 2 min and dissociation was observed for 5 min. Then, the binding signal to C3b was determined for each construct by injecting each protein at a concentration 10-fold higher than the respective measured K_D (100 µM FH18-20, 60 µM FH18-20(LA), 6 µM FHR-1 and 15 µM FHR-1(SV)) for 2 min, followed by 5 min dissociation monitoring. Finally, C3 (1 µM) and each construct (at a concentration 10-fold higher than the K_D) were co-injected for 2 min and dissociation was observed for 5 min. For regeneration, 1 M NaCl and buffer were flowed for 0.5 and 2 min, respectively, after each protein injection. All experiments were performed in duplicate, at 25°C in PBS containing 0.005% Tween20 and at a flow rate of 25 µl/min.

Inhibition of C5 binding to C3b

Inhibition of C5 binding to C3b was performed by SPR in a similar manner as described for the inhibition of C3 binding to C3b, with only a few changes. For this experiment, 1500 RUs of biotinylated C3b were immobilized on the streptavidin chip and a concentration of 0.15 µM C5 was used instead of C3. Additionally, the single mutants of FH18-20 were also tested using concentrations 10-fold higher than their K_D values (100 µM FH18-20(SA) and 60 µM FH18-20(LV)). As a control, the same measurements were performed with 100 µM FH19-20.

Guinea pig erythrocyte haemolysis assay

Guinea pig erythrocytes were supplied in Alsever's solution (Davids Biotechnologie, Regensburg, Germany) and washed with PBS. For experiments with desialylated erythrocytes, 400 µl cell suspension (~ 1.3 x 10^9 cells / ml) was incubated with 150 U Neuraminidase for 1 h at 37 °C. A mixture of 10 µl of cell suspension (desialylated or untreated), 10 µl of analyte solution and 20 µl NHS diluted in PBS and supplemented with Mg-EGTA (final serum and Mg-EGTA concentration: 40 % and 5 mM, respectively) was incubated for 30 min at 37 °C. The reaction was then stopped by adding 120 µl ice-cold PBS supplemented with 5 mM EDTA. Afterwards the samples were spun down and the absorbance of the supernatants was measured at 405 nm. Control reactions included cells incubated with HIS, glycine buffer at the highest used concentration in the assay or EDTA at 5 mM final concentration. Haemolysis was calculated as the quotient of measured absorption of the sample and total lysis in water (100% reference).
